# The power of shared positivity: organizational psychological capital and firm performance during exogenous crises

**DOI:** 10.1007/s11187-021-00506-4

**Published:** 2021-06-06

**Authors:** Ann-Christin Grözinger, Sven Wolff, Philipp Julian Ruf, Petra Moog

**Affiliations:** Chair for Entrepreneurship and Family Business, University of Siegen, Siegen, Germany

**Keywords:** Crisis, Individual psychological capital, Organizational psychological capital, Performance, Creativity, Organizational citizenship behavior, L20, L25, L26

## Abstract

This study examines the influence of organizational psychological capital on the performance of small and medium-sized companies (SMEs) during crises. We argue that SMEs use their intangible resources to cope with difficult situations such as the COVID-19 pandemic. Therefore, we investigate how organizational psychological capital impacts performance and creative innovation through such intangible resources, namely, organizational citizenship behavior, solidarity, and cooperation. Methodologically, we combine structural equation modelling and regression analysis on a dataset of 379 SMEs. Our results support the notion that organizational psychological capital positively influences creative innovation of SMEs and thus performance during crises. Our research contributes to the organizational behavior literature by showing that psychological resources of SMEs can strengthen performance in times of crisis and help to prepare for future ones.

## Abstract (plain language summary)

The COVID-19 pandemic has impacted our economic system tremendously and continues to be a threat, especially for small and medium-sized companies (SMEs). Our research shows that a shared positive thinking, namely, Organizational Psychological Capital, can be used by SMEs to counteract the negative effects of exogenous crises, such as the COVID-19 pandemic. Using a dataset of 379 SMEs, we highlight that organizational psychological capital positively affects creative innovations of SMEs and leads to better performance. Thus, the principal implication of our study is the fact that SMEs should place a stronger emphasizes on their organizational psychological capital and try to stay positive, as it will help them to come up with innovative ideas coping with the effects of the crisis, ultimately increasing their performance and survival rate.

## Introduction

In early 2020, the world was hit by a global pandemic caused by the coronavirus (Bacq et al., 2020). This pandemic disturbs and challenges society and established economic systems throughout the world (Li & Tallman, 2011). In addition to overburdened healthcare systems, a recession is threatening the global economy (Global Data PLC, 2020), posing a major challenge for companies now and in the near future. Measures, such as social distancing to prevent the virus from spreading rapidly (Glass et al., 2006) and lockdowns in many countries changed the reality not only for society but also for organizations (Kuckertz et al., 2020). Due to the COVID-19 pandemic and resulting governmental regulations, many companies had to restrain their business activities completely and revenues dropped significantly. Rapid change is needed for companies, industries, and markets to survive, as uncertainty increases and financial security decreases (Beliaeva et al., 2020). Companies are forced to act, to secure their employees, their market position, their performance, and ultimately their survivability. Especially small and medium-sized companies (SMEs) are struggling with the situation as they are more vulnerable to shocks and their long-term effects (e.g. Cucculelli & Peruzzi, 2020), face difficulties in accessing financial capital (Karlsson, 2020), and oftentimes lack the physical resources to pull through such times of adversary. These difficulties of SMEs are also highlighted by the resource-based view (Barney, 1991; Crook et al., 2008) and the liability of smallness (Alrich & Auster, 1986; Fackler et al., 2013), both arguing that due to their larger size, publicly-traded companies outperform their smaller and privately operated counterparts.

Especially during crises, when resource scarcity is even more evident, it is necessary to secure one’s performance in order to survive. However, research into the drivers and factors influencing performance in SMEs is fragmented (Davidsson et al., 2010), which also holds true for the limited research results that are concerned with performance of SMEs in crises situations (Cowling et al., 2018). While it is widely acknowledged that the leaders of SMEs with their personal capabilities and characteristics can influence the performance of the companies (Anderson & Reeb, 2003; Hansen & Hamilton, 2011; Smallbone et al., 1995; Wiklund & Shepherd, 2003), Cowling et al. (2018) find that in the period immediately following the global financial crisis (2008–2010) such characteristics of entrepreneurs only showed a very minor influence on the performance of their companies. Furthermore, research focusing on whether cost cutting or revenue generating measures secure performance during crises shows that SME actions are very diverse and result in different performance outcomes (Beliaeva et al., 2020; Collett et al., 2014; Kottika et al., 2020; Latham, 2009; Smallbone et al., 2012). Additionally, Helton and Head (2012) report that the psychological issues like stress and anxiety that arise from crises situations negatively influence performance, which raises the question on why some SMEs seem to be more “immune” to this negative consequences accompanying the current situation. The above-mentioned facts raise several unanswered questions. What can SMEs do to overcome the COVID-19 pandemic and future, similar situations? What unique resources do they have and may utilize to maintain or even increase their performance while their very existence is being threatened and why do certain SMEs perform better than other SMEs?

To answer some of these questions, recent research focused on the intangible resources which can provide companies with sustained competitive advantages (Luthans & Youssef, 2004). Next to the known concepts of human capital (Delery & Roumpi, 2017) and social capital (Lins et al., 2017), organizational psychological capital (OPC) which represents the positive psychological state of an organization was introduced by McKenny et al. in 2013. OPC is derived from the individual psychological capital (PsyCap) (Luthans et al., 2007; McKenny et al., 2013) which is rooted within the positive organizational behavior research (POB) (Luthans, 2002a), and strongly related to psychological studies (Luthans, 2002b), investigating the influence of psychological resources within humans on the performance of firms (Luthans, 2002a). Methodically, OPC is derived and conceptualized on a collective level of analysis, while PsyCap is measured on an individual level. Besides the level of measurement, the constructs are very similar, consisting of four dimensions, namely, hope, resilience, optimism, and efficacy (Luthans & Youssef-Morgan, 2017; Newman et al., 2014). First empirical evidence by McKenny et al. (2013) shows that OPC can be considered to be an intangible resource in organizations. This effect is argued to be strongest in SMEs (McKenny et al., 2013) and family businesses (Memili et al., 2013) and might give those companies an edge over larger publicly traded companies especially in difficult times (Memili et al., 2013).

While a lot of recent crises literature focused on the resilience of companies (Williams et al., 2017), there are few studies about individual PsyCap in connection to crises (e.g., Raja et al., 2020) and so far, to the best of our knowledge no studies about the influence of a collective level of OPC during a crisis. This is surprising as both concepts contain resilience as a dimension (Luthans et al., 2007; McKenny et al., 2013) and deal with positivity, which is, especially in difficult times, extremely important (Memili et al., 2013). Of course, overcoming a global crisis does not solely depend on the psychological state of a company. As already mentioned, especially SMEs, due to the constraints of physical resources, depend on the loyalty and support of employees and external stakeholders (Bin & Edwards, 2009; Ogawa & Tanaka, 2013) as well as mobilization of creativity within the company (Kuckertz et al., 2020; Williams et al., 2017). Due to PsyCap being closely related to the well-being of employees (Aveyet al., 2010a) and resulting employee behavior (Avey et al., 2011), we argue that OPC is actively fostering the mentioned means to overcome a crisis for SMEs. Therefore, while OPC is not the only factor, which helps to cope with a crisis, it is one of the few fundamental states of a company which helps to boost behavior, favoring a survival of a company. Accordingly, we argue that a high OPC helps companies to maintain a positive attitude throughout the current COVID-19 pandemic and ultimately increases the performance of those. We specifically ask the questions:

- *Does the organizational psychological capital of SMEs influence the company’s performance during the COVID-19 pandemic?*

Our study contributes to literature in a threefold way: Foremost, we add to the entrepreneurship literature by addressing the question of why some SMEs perform better during difficult times than others. We apply a positive psychology perspective (Gable & Haidt, 2005) and introduce the concept of OPC as a unique resource of SMEs, enhancing performance, influencing creative innovation and other desirable firm behaviors like organizational citizenship behavior (OCB), solidarity, and cooperation in the COVID-19 context. Besides that, we find a direct and indirect impact of OPC on the performance in this adverse circumstance. Thus, we create new insights on how SMEs can survive situations like the COVID-19 pandemic, even though they face certain resource constraints. Such knowledge is extremely important for SMEs currently experiencing crisis and also in the future. Research has shown that individual PsyCap can actively be developed through short training sessions (Dello Russo & Stoykova, 2015). If a higher OPC is able to increase performance of companies throughout situations such as the COVID-19 pandemic, companies should focus on steadily expanding their OPC in order to strengthen their resilience. This would increase the ability to deal with the current situation and the resistance to future crises. Second, we add to the small body of research on OPC and add empirical results. We show that the PsyCap is a construct, which can be lifted to the organizational level (namely, OPC). We also test the connection of OPC of SMEs on performance, creativity, desirable company behaviors, and cooperation during crises. Third, most of the research in crisis management has focused on times before the crisis (preparation, causes) and the aftermath of a crisis (Williams et al., 2017). As the COVID-19 pandemic is still ongoing, we have the unique opportunity to investigate how SMEs behave and deal with an enduring crisis, by showing that the collective level of positivity within these companies can become a viable factor in such circumstances.

## Theoretical background

### Organizational psychological capital as an intangible resource in SMEs

In order to survive times of crises, companies can make use of their unique resources (Sirmon & Hitt, 2003). According to Barney (1991) and the RBV, competitive advantages and increased performance may result by leveraging a company’s unique resources (Crook et al., 2008). The RBV favors big publicly traded companies in times of crisis, as their access to financial and human resources is substantially better. This is supported by empirical evidence that especially SMEs as well as younger firms suffer throughout crisis, as they are more vulnerable to shocks and the following long-term effects (e.g. Cucculelli & Peruzzi, 2020). In line with the concept of liability of smallness (Alrich & Auster, 1986; Fackler et al., 2013), SMEs usually face constraints accessing financial capital (Karlsson, 2020) which is especially critical to situations like complete shutdowns as experienced throughout the COVID-19 pandemic. Many SMEs rely on their regular cash flow to finance their operations, which means that they depend on their revenues to survive (Runyan, 2006).

However, certain researchers stress that in our modern world, traditional resources including financial, physical, and technological capital may no longer be sufficient to provide companies with a competitive edge and that rather intangible resources like human capital (Crook et al., 2008), social capital (Arregle et al., 2007), and psychological capital (PsyCap) (Luthans, 2002a, b) entail the potential to increase or strengthen performance of companies (Luthans & Youssef, 2004). The latter, although initially defined as an individual construct, is lately receiving attention at higher levels of analysis as there is growing evidence, that PsyCap also exists in collective structures (Broad & Luthans, 2017; Clapp-Smith et al., 2009; Dawkins et al., 2015, 2018). PsyCap on a company’s level (i.e., OPC) was introduced by McKenny et al. (2013).

Both constructs, PsyCap and OPC, are rooted in POB research (Luthans, 2002a; Wright, 2003) and thus related to positive psychology (Gable & Haidt, 2005; Luthans, 2002b). They also clearly distinguish themselves from other constructs such as emotions or the Big Five personality dimensions (Barrick & Mount, 1991). The Big Five personality traits are considered to be characteristics which are very stable in their nature, so that they tend to change rather little over the course of a lifetime and are therefore considered to be personality traits (Luthans et al., 2007). PsyCap and OPC, however, are psychological states, which are subject to change. POB focuses on positive psychological resources and abilities within humans, which can be improved and managed, ultimately influencing performance (Luthans, 2002a). By considering a cost–benefit view POB differs from positive psychology (Wright, 2003), which only focuses on the positive psychological abilities and resources within individuals (Gable & Haidt, 2005) not taking potential gains into consideration. PsyCap was introduced to the management literature in the early 2000s (Luthans, 2002a, b) with a broad body of studies published on the topic (for a comprehensive overview, see Luthans & Youssef-Morgan, 2017).

Lately researches consider that PsyCap exists within collective structures (e.g., group, collective, and organizational level) (Broad & Luthans, 2017; Clapp-Smith et al., 2009; Dawkins et al., 2015, 2018; McKenny et al., 2013; Memili et al., 2013). As OPC does not perform perfectly isomorphic (Kozlowski & Klein, 2000) to PsyCap, which means that the sum of the individual PsyCap values of a group does not necessarily reflect the organizational level of the construct, adjustments for the collective level are required (Luthans & Youssef-Morgan, 2017). In their article McKenny et al. (2013) conceptualize OPC using the referent-shift model according to Chan (1998). To provide scientific rigor, they validate the OPC construct according to the framework for validating multilevel constructs by Chen et al. (2004). In this regard we follow the approach of McKenny et al. (2013) and base our definition on individual PsyCap by Luthans et al. (2007). We define OPC “ […] as the organization’s level of positive psychological resources: hope, optimism, resilience, and confidence [i.e. efficacy]” (McKenny et al., 2013, S. 157) and thus consider it to reflect the organization’s positive psychological state.

We define the four dimensions on the company level as follows: The idea of organizational hope draws directly from the concept of hope by Snyder et al. (1991) and embodies the positive state of motivation within an organization. It is expressed in a common goal-oriented dynamic with a shared belief that the objectives can be achieved in different ways (Luthans et al., 2007). Thus, organizations showing a high level of organizational hope are able to develop and share several company-related goals, which contain both, a long-term and a short-term horizon, and share the common perception that these objectives can be achieved in a multitude of ways (Hmieleski et al., 2015; McKenny et al., 2013; Snyder et al., 1996). The concept of organizational efficacy is based on the work of Bandura (1997, 2012). It is expressed through a shared trust of the company in its own abilities and cognitive resources, which are necessary to perform certain tasks, and the belief that these can be mobilized (Luthans & Youssef, 2004). This positive assessment of the companies abilities is reflected in a shared confidence in the capabilities of the organization (Stajkovic & Luthans, 1998). Consequently organizations that show a high level of organizational efficacy rely strongly on their capabilities and thus are able to pursue more ambitious goals than companies low in this psychological resource (Bandura, 2012; McKenny et al., 2013). Rooted in clinical psychology (Masten, 2001; Masten & Reed, 2002), the concept of organizational resilience characterizes the psychological capability that enables the organization to overcome setbacks and crises jointly and to recover, thereby improving its performance over the initial level (Luthans, 2002b). Thus, companies high in organizational resilience are better able to “bounce back” from adverse developments as they tolerate those developments and thus constructively deal with such situations by aiming to solve the situation (Luthans et al., 2006; McKenny et al., 2013). In contrast the psychological resource of organizational optimism represents the organizations shared positive reasoning that assigns positive developments to lasting and persistent triggers and negative events to local, transient, and situation-specific occurrences (Luthans & Youssef, 2004). Thus companies with a high level of organizational optimism use positive reasoning when facing obstacles, expecting positive outcomes (McKenny et al., 2013; Scheier et al., 2001). Those four positive organizational resources, when combined represent the higher-order construct of OPC (Luthans et al., 2015).

However, little is known on how psychological resources on an organizational level (e.g., collective structures) influence the SMEs ability to deal with the quickly changing reality during crisis and how those companies can use positive psychological resources to survive or even thrive in adverse circumstances. First empirical evidence by McKenny et al. (2013) shows that OPC impacts the performance of large and publicly-traded companies positively. They further argue that the influence of OPC might be even higher in SMEs due to the stronger and more direct influence of each employee working within the company. Thus, further research which considers OPC as an intangible resource is encouraged. Especially in difficult times, it might provide SMEs with a competitive edge increasing their chances of survival (Memili et al., 2013; Sirmon & Hitt, 2003). The potential of OPC becomes even more evident, considering the malleable nature of PsyCap and presumably OPC. As previously mentioned PsyCap can be regarded as a state-like resource, which can be altered (Luthans et al., 2007). Studies have already shown that the PsyCap can change over time (Avey et al., 2010a; Peterson et al., 2011) and that it can be increased through short training interventions (Dello Russo & Stoykova, 2015). This changeability of PsyCap has an immense potential for entrepreneurship and management research, given that a positive influence is exerted on various desirable outcomes such as employee’s performance (Luthans et al., 2010), their behavior (Avey et al., 2011), and attitudes (Larson & Luthans, 2006).

Complementary to McKenny et al. (2013), Pearson and Clair already proposed a psychological view on crisis management in 1998. They argue that individuals and groups play a crucial role in organizational crisis as their coping behaviors (i.e., cognitive, behavioral, and emotional responses) shape the direction of the crisis within the company. Even though crises are considered as negative events, James et al. (2011) suggest that a positive psychological view on crises can enhance the understanding of reactions to crises in organizations. In this context research already shows that positive cognitive responses help to maintain the functioning of an organization in such critical times (Dewald & Bowen, 2010). Besides that, Penrose (2000) shows that the perception of opportunities and thus a positive view on the situation improve the organizations dealing with a crisis. We therefore follow the call to include positive psychology in crisis research by focusing on OPC. To the best of our knowledge, no study has yet examined the possible influence of OPC on a company’s behavior during crisis. First empirical evidence on a possible influence exists for the individual PsyCap of leaders. Milosevic et al. (2017) investigate how Winston Churchill in his role as a country leader leveraged his PsyCap during World War II by analyzing transcripts of his speeches during that time. Results show that leveraging on PsyCap during crisis exhibits the opportunity to activate behaviors to overcome adverse situations.

### Organizational citizenship behavior, solidarity, cooperation, and creative innovation during crises

Besides the role of psychological resources, companies take actions which help them to thrive during crises. Many companies and individuals offered their support to assist those affected most by the situation to overcome those troublesome times. This assistance is usually called prosocial behavior (Jonas, 2012). For example, after hurricane Katrina, which hit the USA in 2005, Rodríguez et al. (2006) found that prosocial behavior was the dominant response for the broad majority. Apart from the psychological literature, a similar construct describing this behavior has been established in the management literature, namely, organizational citizenship behavior (OCB) (Organ, 2018). OCB reflects a set of positive behaviors in the workplace which are not part of the work tasks of the respective employees but rather are taken voluntarily (Podsakoff et al., 1990). Another kind of prosocial behavior that was witnessed in the early research on the effects of the COVID-19 pandemic can be described as solidarity. Companies started to shift their manufacturing focus on products to help contain the virus, sometimes even donation parts of their production (He & Harris, 2020). Evidence focusing on SMEs shows that they widely engage in disaster relief for their community (Bin & Edwards, 2009), proofing that besides governments and globally operation cooperation, also entrepreneurs and SMEs become active (Markman et al., 2019) and take responsibility as they understand solidarity actions as part of their societal mission and also like to return something to the community (Acs & Phillips, 2002). Furthermore, as shown in his very early research on the impact of the COVID-19 pandemic on young and relatively small firms, Kuckertz et al. (2020) find that the companies widely activated their networks to receive support from external stakeholders. In line with this results, Doern et al. (2019) show that the activation of the external network helps SME’s to recover from crisis. Furthermore, they show that a positive perspective of the respective firms also facilitated their recovery, also in the respect that they positively perceived the support of external sources.

Besides the shown behaviors of SMEs, further research suggests that in crisis context those companies react in a more creative way to the emerging opportunities than larger companies (Williams et al., 2017) which can ultimately help SMEs to survive the adverse time (Battisti & Deakins, 2017). These opportunities arise as a result of changing conditions, which becomes evident by the following example in the COVID-19 crisis: People’s consumption habits and needs shift during the pandemic, which lead to an increase in online shopping (Kottika et al., 2020). For stationary retailers and restaurants, the lockdowns presented a major challenge. These companies could not offer and sell their products as usual. It becomes evident that especially SMEs faced great challenges in this regard, as they are often small, independent stationary stores or restaurants that do not have online stores or do not offer a delivery service (Ibn-Mohammed et al., 2021). A large part of SMEs reacted to the governmental restrictions with creative changes in distribution, products, and other areas by implementing online accessibility of their products, delivery solutions, or started to produce products whereas demand increase due to the pandemic (He & Harris, 2020; Welter et al., 2020). Thus the COVID-19 circumstances offered a wide range of opportunities for companies to react in a flexible and creative way by using their bricolage (Kuckertz et al., 2020). This exploitation of opportunities by innovating has been proven to help SMEs survive in crisis circumstances (Mayr et al., 2017).

Creativity is broadly defined as the thinking and generating of novel ideas by individuals or groups, whereas innovation represents the successful implementation of such an idea (Amabile, 1988; Heunks, 1998; West & Farr, 1996). Thus creativity forms the basis for innovation in companies and both are part of the same progress (Amabile & Pratt, 2016). Since the creativity and innovation process during the COVID-19 pandemic was likely very rapid and integrative in the companies, and as in a general sense both concepts are intertwined in the process (Amabile & Pratt, 2016), it would be difficult to clearly separate the two constructs in our study; thus we consequently use the term creative innovation in the following.

In the RBV it is commonly accepted that the innovation ability of companies strongly depends on their resource base and their ability to make use of them (Kusunoki et al., 1998). Hitt et al. (2001) stress that intangible resources in companies will help to establish a stronger competitive advantage than other resources, as those are difficult to copy for the competitors. Consequently, we theorize that the OPC of companies, which is considered to provide especially SMEs with an intangible resource that can lead to a competitive advantage (McKenny et al., 2013; Memili et al., 2013), will help them to cope with the COVID-19 crisis. In the following, we hypothesize why OPC increases the OCB, solidarity, and cooperation of SMEs during times of crisis, ultimately leading to a higher creative innovation, thus increasing performance during a crisis.

## Hypotheses development

### Organizational psychological capital and its influence on organizational citizenship behavior, cooperation, and solidarity

In his research, Jonas (2012) shows that prosocial behavior in general increase in the event of a crisis. On an organizational level, there is the concept of organizational citizenship behavior (OCB) which reflects prosocial behavior of employees. OCB is defined as “the maintenance and enhancement of the social and psychological context that supports task performance” (Organ, 1997, S. 91). It describes activities taken by members of organizations that benefit their organization or other individuals in this organization without getting anything in return. According to Avolio and Gardner (2005) in turbulent times, companies need to create an excited and motivated workforce to ensure success. This is possible by using intangible resources such as PsyCap, social, and human capital (Arregle et al., 2007; Crook et al., 2008; Luthans & Youssef, 2004). Avey et al. (2008) and Gooty et al. (2009) therefore investigated how the positivity of employees, namely, PsyCap, influences this prosocial behavior or precisely OCB of employees. Both studies found empirical evidence that PsyCap and OCB are positively related. A study by Norman et al. (2010) supports this notion. They found empirical evidence in their sample of 199 working adults that a higher PsyCap leads to more OCB activities within the company, thus arguing that a higher PsyCap in employees might foster desirable work behaviors which are not part of the job description but are rather altruistic in nature. In accordance with the literature, we apply these findings for the individual level to the organizational level of analysis and theorize that OPC influences the OCB of employees during crises.

In line with this social perspective, we also believe that OPC fosters the solidarity which the companies show. The COVID-19 pandemic can be considered as one of the grand challenges humanity is facing right now (Howard‐Grenville, 2021). To resolve those grand challenges, the common view is that rather governments of the world’s leading countries and large multinational cooperation’s will make the difference in dissolving these. It is clear that those big players are crucial in this matter; however Markman et al. (2019) stress that it’s also worthwhile to consider individuals, groups, and small ventures in this equation, as they are also able to contribute to the resolving or mitigate the suffering. In fact there is evidence that after floods following the hurricane Floyd in North Carolina in 1999, rather small companies engaged in local disaster relief (Bin & Edwards, 2009). Especially SMEs that are usually embedded in their local community (Backman & Palmberg, 2015) thus are likely to engage in acts of solidarity. On the one hand, the companies understand this solidarity actions as their obligation through the implicit social contract; on the other hand, they generate something worthwhile for society based on the philanthropic idea of wanting to give something back (Acs & Phillips, 2002). Thus SMEs adopt a social perspective, fostering their connections to the community and adopting social practices (Bin & Edwards, 2009). He and Harris (2020) report that such behavior could also be witnessed during the COVID-19 pandemic. For example, some companies which are active in manufacturing started to reorganize and produce goods that were needed to prevent the pandemic from spreading, like disinfectant and protective clothing, or to save lives by producing urgently needed ventilators. However to date we know very little about factors that facilitate such acts of solidarity from SMEs when society is confronted with grand challenges (Markman et al., 2019) like the recent crisis. We hypothesize that OPC, as psychological resources, influence the solidarity of SMEs as solidarity on a collective level suggests an advanced level of systems thinking that promotes the overall well-being (Hogan, 2020).

Last, we believe that OPC fosters the engagement in cooperation with external stakeholders during the COVID-19 pandemic. In their study on the reactions of start-ups during the pandemic, Kuckertz et al. (2020) find that those young and rather small companies activate their networks with stakeholders. The ability of SMEs to understand their connections and to activate their network is useful in times of crisis in order to use their resources effectively and thus to be able to achieve their goals (Battisti & Deakins, 2017). In crisis circumstances, one can assume that the main goal for most companies is to secure their survival. SMEs start to engage more in cooperation’s with external stakeholders during crises due to several reasons. First, as SMEs are confronted with limitations in resources due to their small size (Bin & Edwards, 2009; Fackler et al., 2013; Ogawa & Tanaka, 2013), they try to compensate this disadvantage by cooperation (Jones & Macpherson, 2006). Furthermore, their embeddedness in the community stimulates the need to help (Bin & Edwards, 2009) and to fight the grand challenges. However, they know that they lack the knowledge, resources, and skills to counteract the crisis alone. Thus they engage in collective actions (Markman et al., 2019). Second, crisis circumstances are characterizes by quick dynamic changes which are inherently connected with a high degree of uncertainty and a lack of information (Herbane, 2010; Vargo & Seville, 2011). This leads to the need to quickly make complex decisions and to adopt to the changing uncertain circumstances, as the companies’ survival depends on that (Latham, 2009). In such situations the gathering of information is crucial and SMEs leverage on their cooperation with different stakeholders to collect more supporting information and knowledge about the situation in order to make better decisions (Jones & Macpherson, 2006; Mayr et al., 2017). Third and in line with empirical evidence we believe that SMEs also turn to stakeholders for emotional support during a crisis, as a feeling of “we are all in this together” arises (Doern et al., 2019; Wall & Bellamy, 2019).

In summary all these reasons have a common objective, to face the adversities created by the crisis and to cushion the impact of its effects. As OPC, and its underlying dimensions, reflect a company’s positive psychological state and a shared level of agency (McKenny et al., 2013) which increases their motivation to achieve (higher) goals (Newman et al., 2014) and their believe in the abilities to mobilize the necessary resources, to bounce back and work harder in order to overcome adversity (Avey et al., 2011), we theorize the following relationships:

- *H1: The greater the degree of organizational psychological capital, the greater will be the extent of organizational citizenship behavior, solidarity, and joint activities (cooperation) during crisis.*

### Organizational psychological capital and its influence on creative innovation

The ability to discover new opportunities can be crucial for SMEs in times of crisis (Battisti & Deakins, 2017). In the RBV this exploitation of opportunities depends on whether companies can mobilize their resources and react flexibly (Pal et al., 2014). In literature such entrepreneurial opportunities have been widely linked with creativity (e.g. Hansen et al., 2011). Furthermore, it has been shown that in order to stay viable and healthy in difficult times, SMEs react more creatively than large companies (Williams et al., 2017), and innovation is the key to survive in such circumstances (Mayr et al., 2017). During the COVID-19 pandemic, many opportunities were created, giving SMEs the chance to respond creatively (Kuckertz et al., 2020). The question of what factors in SMEs drive creative innovation during times of crisis has to our knowledge not been discovered yet. Amabile and Pratt (2016) recently revised their componential model (Amabile, 1988) to find that creativity and innovation are closely interlinked. They also propose four psychological driving factors for the process on an individual and organizational level, which they believe are analogues to each other on both levels: “a progress loop; meaningful work (and a related construct, work orientations); affect; and new insights into motivation” (Amabile & Pratt, 2016, S. 166). Their argumentation especially empathizes on motivation as a driver which is, according to the authors, closely related to the self-efficacy concept of Bandura (1997). However in a positive psychology view, not only the psychological resource of efficacy is related to motivation but rather the core construct of PsyCap with the other three psychological resources of hope, resilience, and optimism (Peterson et al., 2011). Studies show that individual PsyCap predicts creativity in a direct relationship (Rego et al., 2012; Sweetman et al., 2011). In addition, mediation effects of PsyCap in the context of creativity have been displayed (Huang & Luthans, 2015). Furthermore, there is initial evidence by Luthans et al. (2011) that PsyCap shows a positive relationship with individuals problem-solving ability and reported innovation. Research dealing with theories of social comparison suggests that through workplace interactions, the members of organizations align to a common level of positivity and agency (e.g., Salancik & Pfeffer, 1978; Sullins, 1991). First evidence, that OPC is also connected to creativity and innovation in companies, is given by Wu and Chen (2018). They examined outcome factors for the collective PsyCap (collective level of analysis) and found a positive relationship between the collective level of PsyCap and the creativity of the groups. In light of the widely accepted fact, that human capital provides a critical resource in terms of innovation and creativity (Barney, 1991; Hitt et al., 2001), by recognizing opportunities (Lumpkin & Lichtenstein, 2005) and in line with Amabile and Pratt (2016), we consider OPC as one of the main drivers behind creative innovation in times of crisis. We therefore hypothesize:

- *H2: The greater the degree of organizational psychological capital, the greater will be innovative activity during crisis.*

### Organizational citizenship behavior, cooperation, and solidarity and their influence on creative innovation

Besides the psychological resources that presumably support the creativity and innovation process, Amabile and Pratt (2016) propose that the employees perceived meaningfulness of work represents another factor. Meaningful work in this context can be described as work that is perceived as positive and significant (Rosso et al., 2010). When individuals or groups consider the solving of a problem to be important, their creativity increases in order to contribute to the resolution of the problem (Staw, 1990). Put into the context of the COVID-19 pandemic as a grand challenge for humankind (Howard‐Grenville, 2021), we believe that the resolving of this pandemic is considered as an important problem and thus SMEs would perceive working on problem resulting ideas as meaningful work. We already theorized on why it is likely that SMEs in this recent global crisis would want to engage in helping to resolve or alleviate the pandemic and how this facilitates prosocial behaviors like solidary actions as well as their OCB. However, it might be added that in this respect solidarity at a group level implies a groups joint effort to resolve a problem (Hogan, 2020), whereas OCB shows links to perceived meaningfulness of work (Lam et al., 2016) both ultimately increasing the creative innovation output in SMEs during a crisis.

Besides that, we propose that also cooperation with stakeholders during the recent crisis impacts the creative innovation in SMEs. Due to resource scarcity, which implies a lack of resources and capabilities in SMEs, innovations can lead to the companies being overstretched (Acs & Audretsch, 1988). Thus, to develop and realize innovation, SMEs frequently engage in cooperation with external partners (Shan, 1990). Regarding the effects of such collaborations on the yield of innovation in SMEs, there are mixed findings. On the one hand, it should be noted that SMEs, due to their size, often find themselves in a weaker negotiating position than the larger cooperation partners and therefore have to accept poor conditions when sharing the returns of innovation (Rosenbusch et al., 2011). On the other hand, positive effects are expected in most cases (Rosenbusch et al., 2011), as the bundling of internal and external resources can deliver promising results (Tyler & Steensma, 1998). Gathering information from external stakeholders to generate knowledge is therefore crucial to generate ideas quickly (Andries & Czarnitzki, 2014; Wall & Bellamy, 2019), as the COVID-19 pandemic is accompanied by a need for rapid action. Further studies conducted in a crisis context show that collaborations are an important factor for SMEs to recover from crises. In summary, as important information and knowledge can be gathered (Wall & Bellamy, 2019), their bricolage can be mobilized (Kuckertz et al., 2020) to counteract negative developments, and to join efforts to collectively face challenges (Markman et al., 2019), we hypothesize that increased collaboration with multiple stakeholders will positively influence creative innovation by SMEs during the COVID-19 pandemic.

- *H3: The greater the extent of organizational citizenship behavior, solidarity, and joint activities (cooperation) in SMEs, the greater will be creative innovative activity during times of crisis.*

### Creative innovation and its impact on performance during crises

The logical consequences of the positive cognitive responses described before are behavioral reactions. Especially positive behavioral reactions promote progress in crises. Companies which can adapt to the changed environment, and introduce compatible routines, will most likely do well (Lengnick-Hall & Beck, 2005). As the characteristics of SMEs like flat hierarchies, short communication paths, and quick decision-making power provide them with a high degree of flexibility, they can adopt to the changing circumstances posed through the crisis in a faster manner than their large counterparts, which has a positive impact on their innovation potential. This way they can counteract their limited resource basis in such times (Nooteboom, 1994; Vossen, 1998). Regardless to their resource constrains, SMEs often successfully innovate (Rosenbusch et al., 2011). Research into crises has already shown that in times of uncertainty, SMEs react with a combination of cost-reducing and performance-generating measures, focusing on the latter (Smallbone et al., 2012). However it has to be noted that SMEs, usually due to their smaller size, have a limited scope in terms of cost-cutting measures (Latham, 2009). This is confirmed by Kuckertz et al. (2020) with regard to the COVID-19 pandemic. They show that young, relatively small companies also seek such a balance but do not engage in huge cost-cutting and retrenchment actions. On the one hand, they focus on profitable and value-generating activities and abandon loss-generating activities temporarily. In addition, they show that the companies surveyed increasingly discover new opportunities to solve problems related to the crisis. This is not surprising as engaging in creative innovations offers SMEs a chance to counteract the consequences of crises. Schumpeter (1934) stresses that innovations provide companies with the opportunity to gain revenues as a temporary monopoly can be created. Thus, considering the flexibility of SMEs, they can move fast to secure those revenues at least for a limited amount of time. Additionally, Porter (1980) argues that niche markets, in which SMEs often operate, represent a great potential for innovation. Mayr et al. (2017) show that SMEs that use some kind of innovation are more likely to overcome crises through sustainable reorganization. Their research is in line with the results of the meta-analysis by Rosenbusch et al. (2011) which show that innovation has a positive effect on the performance of SMEs in non-crises situations. We apply those findings to crises situations and hypothesize:

- *H4: The greater the extent of creative innovation during times of crisis in SMEs, the better will be the performance of SMEs during this time.*

### Organizational psychological capital and its influence on performance during crises

In times of crisis, SMEs are often confronted with very limited resources and must therefore try to use their unique competitive advantages to secure their market position (Beliaeva et al., 2020). As already shown, OPC, which can be expressed as a positive psychological state of an organization and thus represents a positive psychological perspective, can be such an unique advantage in SMEs (McKenny et al., 2013). Psychological research suggests that anxiety and stress resulting from crises situations can disturb ideal functioning and thus lower the performance (Helton & Head, 2012). As PsyCap, and consequently OPC, fosters the ideal functioning of individuals (Avey et al., 2008; Avey et al., 2010a ; Luthans et al., 2007) and organizations, we argue that OPC can help to understand how especially SME`s mobilize their resources when facing adversity, which positively impacts the performance of this companies in a crisis. For the individual level PsyCap, various studies already showed that it has a positive influence on the performance of the employees (Avey et al., 2008, 2010b; Luthans et al., 2005, 2008; Peterson et al., 2011). Researchers explain this relationship in such a way that individuals high in PsyCap pose more resources they can activate in order to achieve their goals (Hobfoll, 2002) and thus increase their performance (Newman et al., 2014). However, as individual PsyCap cannot represent the state of a company in general, research started to consider PsyCap on higher levels of analysis to explore the potential that is entailed in such considerations. For this purpose PsyCap was conceptualized at the collective, group, team, or organizational level (Luthans & Youssef-Morgan, 2017) using Chan’s (1998) referent shift model logic. As with the individual PsyCap, possible links to performance of the collective level constructs were examined. In one of the early empirical studies on the group level PsyCap, Clapp-Smith et al. (2009) showed that there is a positive relationship between the group level PsyCap of employees and their performance. In line with this, Mathe et al. (2017) showed that the collective PsyCap of employees in quick-service restaurants has a direct relationship with service quality and revenue of the respective restaurants. Furthermore Peterson and Zhang (2011) showed that the collective PsyCap of top management teams is significantly positively related to business unit performance.

Regarding the organizational level of PsyCap, there is also first evidence which indicates a positive relationship between a company’s level of OPC and its performance. Elaborating a word list of OPC and using computer-aided text analysis, McKenny et al. (2013) examine public CEO letters to shareholders from large publicly traded companies. They find a positive relationship between the OPC and the performance of those companies. However, since the OPC was only accountable for an additional 0.3%, when controlling for past performance of the respective company, they suggest that this relationship might be stronger in smaller and privately held companies and called on research to explore this relationship in greater detail. We follow this call and use survey data to model this relationship. We therefore expect a similar relationship between OPC and performance of SMEs in times of crisis.

- *H5: The greater the degree of organizational psychological capital during times of crisis in SMEs, the better will be their performance during this time.*

**Fig. 1:**
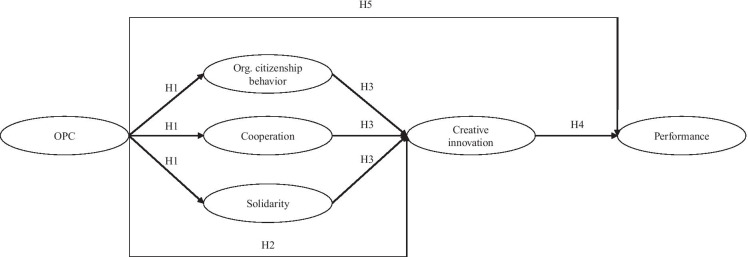
Hypotheses

Figure 1 shows an overview of all hypotheses and their presumed relationships.

## Method

### Dataset

The sample we use to test our hypotheses was collected in an online survey conducted in Germany at the end of July 2020. We decided to focus on one country in particular as of the different dissemination rates and diverse measures taken to deal with the COVID-19 crisis in the individual countries (WHO, 2020). We contacted 20,000 companies in Germany by e-mail and asked them to participate in the survey. The contacts were taken from the Amadeus database (Buerau van Dijk, 2020) with the following requirements in place: The company had to have existed on the market for at least 2 years and had to have a minimum of two employees. We set these restrictions in order to exclude self-employed individuals and nascent start-ups from the analysis. We subjected the data obtained from the survey to a detailed analysis and excluded cases with missing values to perform the various analyses with a constant number of cases. This results in a total sample of 379 cases.

The dataset was tested against a non-response bias by assessing whether the answers of the first respondents differed significantly from those of the last respondents. For this purpose, we compared the first third of the survey responses with the last third of the survey responses and found no significant differences between these groups (Armstrong & Overton, 1977). We can confirm the representativeness of our sample by showing that comparable distributions in terms of industries, company size, company age, and the age of the respondent are present in validated peer reviewed articles about SMEs in Germany (Bongini et al., 2021; Dehlen et al., 2014; Werner et al., 2018). Furthermore, we assured all respondents absolute anonymity and scientific integrity to obtain the most honest responses possible and to prevent a possible social desirability bias (Podsakoff et al., 2003). Additionally, the questionnaire and the cover letter were designed in such a way that the respondents were not influenced by the underlying research question. This was achieved by optimizing the question sequence using pre-tests and by randomizing the question sequence within all question batteries (Podsakoff et al., 2003). Taken together, these measures counteract a possible common method bias (Fuller et al., 2016).

#### Dependent and mediating variables

The main dependent variable in this study represents the performance of the SMEs in the last 6 months, i.e., since the outbreak of the COVID-19 pandemic (Robert Koch Institut, 2020). We do not measure performance on the basis of key measures, because these are often difficult to collect as they involve sensitive company data (Love et al., 2002). We use self-rated assessments of performance relative to competitors, which has been proven to be comparable to key measures (Dess & Robinson, 1984; Eddleston et al., 2007; Love et al., 2002). Respondents were asked to rate on a scale from “much worse = 1” to “much better = 5” how performance in the six areas of (1) sales, (2) revenue, (3) number of employees, (4) net profit margin, (5) market shares, and (6) cash flow has developed over the period of the last 6 months compared to their competitors (Eddleston et al., 2007; Naldi et al., 2007; Smolka et al., 2016; Wiklund & Shepherd, 2003, 2005).

To model the theoretically established relationships from the hypotheses, we used four already validated constructs as mediators. For all four constructs, the respondents were confronted with statements to which they had to indicate their agreement on a 5-point Likert scale from “strongly disagree = 1” to “strongly agree = 5.” They always had to assess the behavior of the company and its employees in the period since the beginning of the COVID-19 crisis. The first construct we used consists of ten questions to measure the OCB in a firm. An example statement for the construct would be: *In order to deal with the challenges since the beginning of the COVID-19 crisis, our employees have often taken time to counsel or mentor a work colleague* (Fox & Spector, 2011). Second, we used a construct consisting of nine questions regarding the solidarity of the firm, proposed by Pérez and Rodríguez del Bosque (2013). An example statement for this construct would be: *During the COVID-19 crisis, our company used parts of its budget for donations and social projects to improve the situation of the most disadvantaged groups in society*. Third, we used a five-question construct to measure the cooperation of the firm with externals, which was proposed by Belderbos et al. (2006). An example statement for the construct would be: *Since the beginning of the COVID-19 crisis, our company has intensified the cooperation with customers (e.g., increased contact, exchange of information, rebooking’s, voucher solutions)*. Lastly, we used a construct of 13 questions to measure creative innovation in the firm proposed by Zhou and George (2001). An example statement for this construct would be*: To deal with the challenges of the COVID-19 crisis, our company has tried new ways to improve quality*.

#### Independent variables

The central influence variable, OPC, is based on the Psychological Capital Questionnaire (PCQ) developed and validated by Luthans et al. (2007). In agreement with the copyright holder, we translated this into German and had it checked by bilingual native speakers. The PCQ contains six items for the four dimensions hope, resilience, optimism, and efficacy and is the most widely used self-report instrument to measure the individual PsyCap (Newman et al., 2014). Using referent-shift model (Chan, 1998), we lifted the PCQ-24 from the individual- to the company-level, which is common practice when measuring PsyCap on a collective level of analysis (Dawkins et al., 2018) This was also done in consent with the copyright holder. The items are measured on a 6-point Likert scale ranging from “strongly disagree = 1” to “strongly agree = 6.” Out of each 6 questions, we calculated mean scores for the four dimensions. Those were used as indicator variables for the construct of OPC. A sample statement for efficacy can be obtained as follows: *In our company we feel confident analyzing a long-term problem to find a solution.*^1^

#### Control variables

To ensure that our analysis is not influenced by unobserved socio-demographic, company-related, or situational factors, we included several control variables in our analysis. First, we included company-related control variables such as the number of employees, as a measure of company size, and the age of the company in the analysis. Both, company size and company age, have already been shown to be related to performance (Karlsson, 2020; Smolka et al., 2016). Furthermore, industry sectors were included in the analysis representing the three main economic sectors. For this purpose, we used the top-level economic classifications of the EU (European Commission, 2011) aggregated them into three sector groups: manufacturing, service, and others. We also included the gender of the respondent (as a dummy variable called female) in the analysis, since the influence of gender on performance is still subject to debating (Kiefer et al., 2020). Finally, we included the extent to which the company is directly affected by the COVID-19 crisis in the analysis, as a strong negative correlation to performance was expectable. Table 1 provides an overview of all used variables and their descriptions. Table 2 shows the minimum, maximum, mean, and standard deviation values for all variables, along with a correlation matrix. The constructs are therefore calculated as mean indices.

**Table 1:** Variable description table

	Variable	Description
1	OPC	Scale consisting of the four PsyCap dimensions according to Luthans et al., (2007): hope, resilience, optimism, and efficacy measured on the company level. Each dimension measured with six questions on 6-point Likert scale ranging from “strongly disagree = 1” to “strongly agree = 6”
2	Org. citizenship behavior	Scale consisting of ten questions based on the proposed ones by Fox and Spector (2011) Rated on a 5-point Likert scale (“strongly disagree = 1” to “strongly agree = 5”)
3	Cooperation	Scale consisting of five questions based on the proposed ones by Belderbos et al. (2006) Rated on a 5-point Likert scale (“strongly disagree = 1” to “strongly agree = 5”)
4	Solidarity	Scale consisting of nine questions based on the proposed ones by Pérez and Rodríguez del Bosque (2013) Rated on a 5-point Likert scale (“strongly disagree = 1” to “strongly agree = 5”)
5	Creative innovation	Scale consisting of 13 questions based on the proposed ones by Zhou and George (2001) Rated on a 5-point Likert scale (“strongly disagree = 1” to “strongly agree = 5”)
6	Performance	Scale consisting of self-assessment relative to competitors since the beginning of the COVID-19 crisis (January 2020) in the following areas: (1) sales; (2) revenue; (3) number of employees; (4) net profit margin; (5) market shares; and (6) cash flow, on 5-point Likert scales ranging from “much worse = 1” to “much better = 5”
7	Employees	Number of employees
8	Firm age	Actual age of the firm
9	Industry — Manufacturing	Dummy equals 1 for the manufacturing industry
10	Industry — Service	Dummy equals 1 for service industry
11	Industry — Others	Dummy equals 1 for other industries than manufacturing or service
12	Female	Dummy equals 1 for females
13	Crisis-affected	Self-assessment whether the company was affected heavy by the COVID-19 crisis on a 5-point Likert scale ranging from “strongly disagree = 1” to “strongly agree = 5”

**Table 2:**
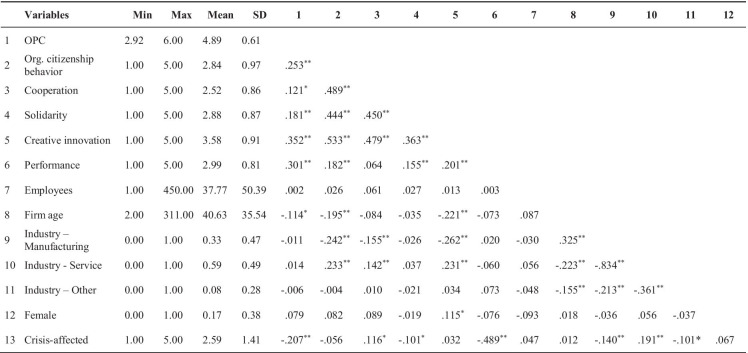
Descriptive statistics and correlation matrix.

Significance levels: ^*^p < 0.05, ^**^p < 0.01 (two-tailed); *N* = 379.

### Data analysis

To analyze the formulated hypotheses, we use a structural equation model in this study. Since the constructs are latent and indirectly measured by indicator variables, by using this method, we are able to integrate these variables into the calculation (Chin, 1998). We are particularly interested in the underlying structure and drivers behind the constructs and therefore use partial least squares structural equation modelling (PLS-SEM) for our analysis, as it is the most appropriate method for this purpose (Hair et al., 2011). We used the SmartPLS software in version 3.3.2. According to the recommendations of Hair et al. (Hair et al., 2016) we have chosen the settings for the calculation algorithm of SmartPLS as follows: path weighting scheme, a maximum of 300 iterations, and the stop criterion at 10^–7^. Bootstrapping with 5000 subsamples as full bias-corrected and accelerated-(BCa) bootstrapping with a two-sided significance test at the 0.05 significance level.

## Results

To inspect the reflective latent constructs, the metrics recommended by Hair et al. (Hair et al., 2019) were calculated and examined. Due to the indicator reliability test, one question had to be excluded from the solidarity scale as its loading was too low. Table 3 lists the composite reliability, Cronbach’s alpha, and average variance extracted (AVE) for the constructs. The values are all within the recommended limits, except for the AVE of the latent variable cooperation. However, this appears not to be a problem, as both the composite reliability and Cronbach’s alpha are well above the threshold values, and thus the convergence validity of the cooperation factor is nevertheless given (Fornell & Larcker, 1981; Hair et al., 2016).

**Table 3:** Cronbach’s alpha, composite reliability, and average variances extracted for reflective measurement models

Construct	Composite reliability	Cronbach’s alpha	AVE	R^2^ (*p* values)
OPC	0.838	0.747	0.567	
Org. citizenship behavior	0.931	0.918	0.576	0.152 (0.000)
Cooperation	0.811	0.716	0.466	0.076 (0.003)
Solidarity	0.890	0.862	0.505	0.045 (0.047)
Creative innovation	0.957	0.951	0.636	0.451 (0.000)
Performance	0.920	0.894	0.662	0.317 (0.000)

To test the discriminant validity of the constructs, we first checked whether the cross-loadings were lower than the indicator loadings, which was the case, thus proving the discriminant validity of the constructs (Chin, 1998). Second, we performed the Larcker test for discriminant validity, which is shown in Table 4. As the square roots of the AVEs are higher than the correlations of the constructs, it also confirms the discriminant validity (Fornell & Larcker, 1981).

**Table 4:** Larcker test for discriminant validity

Construct	OPC	Org. citizenship behavior	Cooperation	Solidarity	Creative innovation	Performance
OPC	**0.753**					
Org. citizenship behavior	0.271	**0.759**				
Cooperation	0.156	0.496	**0.683**			
Solidarity	0.193	0.460	0.453	**0.711**		
Creative innovation	0.358	0.551	0.497	0.390	**0.798**	
Performance	0.325	0.179	0.078	0.151	0.204	**0.814**

Diagonal values in bold are the square root of the AVEs and off-diagonal values are the construct correlations

### Hypotheses testing

**Fig. 2:**
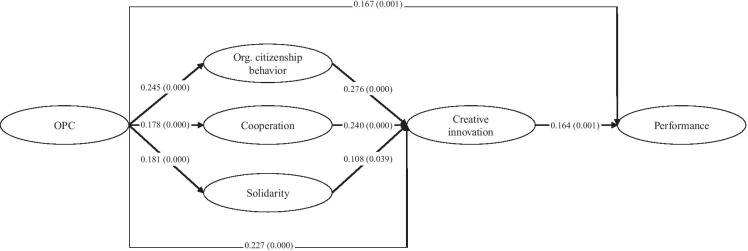
PLS-SEM model

Figure 2 shows our PLS-SEM model, the results, path coefficients, and *p* values. Table 5 gives a more in depths overview, also displaying the t-values, f^2^, and q^2^ effect size.

**Table 5:** Results of hypotheses tests

Hypotheses paths	Hypotheses	Path coefficients	T-values (*p* values)	f^2^	q^2^ effect size	Effect significant
OPC → Org. citizenship behavior	H1	0.245	5.028 (0.000)	0.065	0.033	Yes
OPC → Cooperation	H1	0.178	3.537 (0.000)	0.032	0.012	Yes
OPC → Solidarity	H1	0.181	3.651 (0.000)	0.032	0.014	Yes
OPC → Creative innovation	H2	0.227	4.831 (0.000)	0.081	0.040	Yes
Org. citizenship behavior → Creative innovation	H3	0.276	5.133 (0.000)	0.085	0.040	Yes
Cooperation → Creative innovation	H3	0.240	4.741 (0.000)	0.069	0.033	Yes
Solidarity → Creative innovation	H3	0.108	2.066 (0.039)	0.015	0.007	Yes
Creative innovation → Performance	H4	0.164	3.337 (0.001)	0.031	0.015	Yes
OPC → Performance	H5	0.167	3.253 (0.001)	0.033	0.018	Yes
OPC → Org. citizenship behavior → Creative innovation → Performance		0.011	2.289 (0.022)			Yes
OPC → Cooperation → Creative innovation → Performance		0.007	2.132 (0.033)			Yes
OPC → Solidarity → Creative innovation → Performance		0.003	1.411 (0.158)			No

The control variables are not displayed to provide a better overview. For information on the control variables, see Table 7 in the appendix.

Referring to our hypotheses, we found a positive significant influence of *OPC* on *OCB* (0.245, *p* < 0.001), *OPC* on *cooperation* (0.178, *p* < 0.001) and *OPC* on *solidarity* (0.181, *p* < 0.001), and thus confirm the first hypothesis. Likewise, a significant positive influence of *OPC* on *creative innovation* (0.227, *p* < 0.001) is shown, which confirms the second hypothesis.

For the further connections of *OCB* on *creative innovation* (0.276, *p* < 0.001), *cooperation* on *creative innovation* (0.240, *p* < 0.001) and *solidarity* on *creative innovation* (0.108, *p* < 0.05), significant positive influences were found, as hypothesized in the third hypothesis. *Creative innovation* itself also shows a positive impact on the company’s *performance* (0.164, *p* < 0.01) during the crisis, which supports hypothesis four. Ultimately, the direct significant effect of OPC on the firm’s performance during crisis (0.167, *p* < 0.01), parallel to indirect pathways via the mediators, confirms hypothesis five. For the whole mediation paths of *OPC* via *OCB* and via *creative innovation* on *performance*, we found a significant positive complimentary mediation. Likewise, for the path of *OPC* via *cooperation* and via *creative innovation* on *performance*. The indirect path which includes *solidarity* turned out to be not significant.

### Robustness tests

**Fig. 3:**
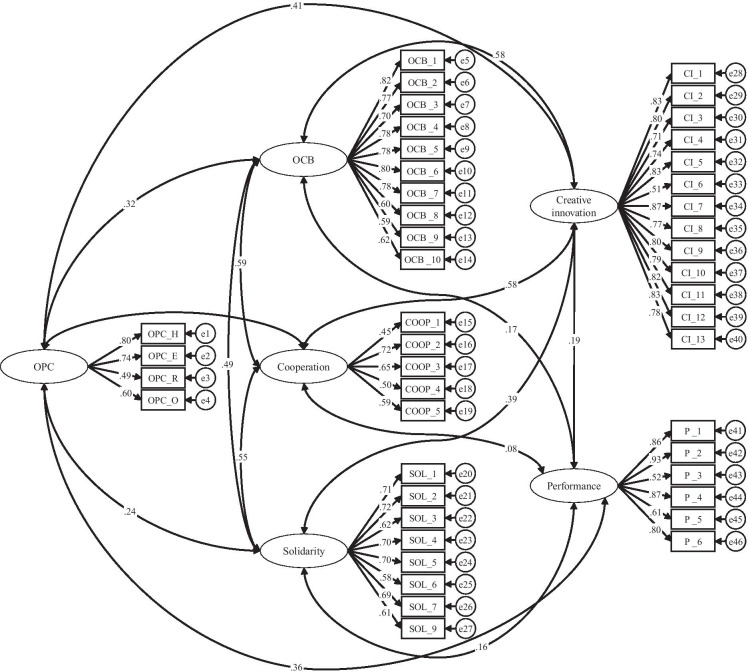
Confirmatory factor analysis. Standardized estimates are shown. *N* = 379. Fit indices: Chi-square = 1912.715 (df = 974 *p* < 0.000), CFI = 0.907, SRMR = 0.057, TLI = 0.901, RMSEA = 0.050, PCLOSE 0.399

To validate our results and to prove the robustness of our findings, we conducted some tests with alternative methodological approaches. First, we performed a confirmatory factor analysis to show how the individual items load on the latent constructs and how the factors separate themselves from each other in our model. We performed this analysis using AMOS with the maximum likelihood discrepancy function. The results of this confirmatory factor analysis are shown in Fig. 3. The confirmatory factor analysis proves that the individual items load well on the latent factors and at the same time the covariance between the constructs is not too high. The model fit indices are all well within the assigned ranges and thus indicate a good model fit (Hu & Bentler, 1999).

For these validated constructs, we further calculated the mean indices for the factors and used them in ordinary least squares regressions to validate our model from the PLS-SEM. The correlations in Table 2 indicate that, apart from the strong negative correlation between the industry dummies, there are no noteworthy correlations. The VIF values for the regression models vary between 3.392 and 3.592 for the industry variables and between 1.026 and 1.604 for all other variables. Thus, multicollinearity can be excluded for our model. The results of these regression analyses are shown in Table 6. Using 5 regression models, we reproduce the same relationships as in our structural equation modelling. We found comparable effect sizes and significances for the relationships, which demonstrate the robustness of our model and the effects.

**Table 6:** OLS regressions-robustness test

Independent variables	Dependent variables				
	Org. citizenship behavior	Cooperation	Solidarity	Creative innovation	Performance
OPC	0.222^***^	0.134^*^	0.165^**^	0.263^***^	0.152^*^
Org. citizenship behavior				0.269^***^	
Cooperation				0.241^***^	
Solidarity				0.094^*^	
Creative innovation					0.169^***^
Employees	0.034	0.062	0.025	− 0.006	0.017
Firm age	− 0.111^*^	− 0.038	− 0.010	− 0.080	− 0.012
Industry — Manufacturing	− 0.107	− 0.116	0.024	− 0.128	− 0.027
Industry — Service	0.121	0.005	0.068	− 0.011	− 0.036
Female	0.063	0.073	− 0.028	0.048	− 0.074
Crisis-affected	− 0.053	0.119^*^	− 0.076	0.060	− 0.455^***^
Observations	379	379	379	379	379
*R*^*2*^	0.141	0.062	0.041	0.432	0.310
*Adjusted R*^*2*^	0.125	0.045	0.023	0.416	0.295
*F*	8.697^***^	3.521^**^	2.265^*^	27.950^***^	20.817^***^
Durbin-Watson	2.075	2.134	1.807	1.899	2.113

Significance levels: ^*^ *p* < 0.05, ^**^p < 0.01, ^***^p < 0.001

Standardized estimation coefficients are reported

## Discussion

The recent COVID-19 pandemic which hit the world in early 2020 poses huge challenges for companies around the globe (Bartik et al., 2020). In line with the RBV (Barney, 1991) and the concept of liability of smallness (Alrich & Auster, 1986; Fackler et al., 2013), especially SMEs, due to their small size and the thus accounted resource constraints, were hugely affected by measures policymakers took to prevent the virus from spreading. Lockdowns of nonessential businesses (Bartik et al., 2020), working from home (George et al., 2020), and measures of social distancing (Glass et al., 2006) became the new normal, leaving many businesses with the challenge of not being able to maintain their performance. With our research, we contribute to the understanding of how SMEs can secure their performance during these difficult times, as research into this issue is scare and fragmented (Cowling et al., 2018). In line with research, we stress that SMEs can perform better in crises by leveraging their unique competitive advantages (Sirmon & Hitt, 2003). By introducing a positive psychology perspective, and thus responding to several calls of POB advocates (Gable & Haidt, 2005; James et al., 2011; Pearson & Clair, 1998; Wright & Quick, 2009), we show that the positive psychological state of SMEs — their organizational psychological capital (OPC) (McKenny et al., 2013) — offers a suitable lens in understanding how some SMEs can use creative innovations on a small scale, positively influencing their performance.

This can also be considered a main finding of our study. Even though SMEs may suffer from certain resource constraints and thus are often expected to underperform especially in times of a resource scarcity, they do have resources which help to mitigate the effects of a crisis. Our results show that OPC is directly and positively related to creative innovation as well as performance. With these findings, we contribute to the ongoing discussion about the factors, influencing performance in SMEs. We show that besides the potential importance of the SME leader’s characteristics (Anderson & Reeb, 2003; Hansen & Hamilton, 2011; Smallbone et al., 1995; Wiklund & Shepherd, 2003), the shared level of OPC within the company — and thus the employees — exhibits great potential for performance in crises circumstances. We thus add that also psychological factors on a company level and not only on an individual level should be considered when trying to understand the various performance outcomes SMEs show during a crisis. We also enhance the results of McKenny et al. (2013). Based on a text-aided analysis, they found a positive relationship between OPC and a company’s performance in shareholder letters. With our survey data, we confirm the robustness of their results showing that the OPC of a company can be considered a competitive advantage. We reason that, similar to the individual PsyCap, companies with a high level of OPC possess more resources that can be activated in order to effectively reach goals (Hobfoll, 2002), which increases the performance of the respective firm.

While previous research has broadly acknowledged that innovation (Rosenbusch et al., 2011) and creativity (Huang & Luthans, 2015; Rego et al., 2012; Sweetman et al., 2011) can enhance an SMEs performance during stable times, we expand this knowledge by proofing that this relationship also holds true for crises contexts such as the COVID-19 pandemic. In respect to the performance and creative innovation relationship in crises, we stress that due to an SMEs flexibility (Nooteboom, 1994; Vossen, 1998), they can quickly engage and implement small-scale creative innovations and thus adopt to the fast changing circumstances arising from the COVID-19 pandemic (Kuckertz et al., 2020). This ability leads to a momentary monopolistic position (Schumpeter, 1934) that positively influences the performance of the SMEs. Our results show that SMEs higher in the shared psychological resources hope, resilience, optimism, and efficacy together forming the higher order construct of OPC engage in more creative innovation and thus can increase their chances of entering the state in which they hold this monopolistic position. This confirms the assumptions made by Amabile and Pratt (2016) in their *dynamic componential model of creativity and innovation* that psychological resources support this process. Thus, our results expand the knowledge about psychosocial factors and their influence on creativity to a collective company level, and by that show, that research in the area of positive psychology on different levels of analysis can provide crucial insight into how creativity can be fostered.

We also examine different intangible factors that could influence creative innovation in crises contexts, which have to our knowledge, not been investigated so far. In global crises situations, besides the government and larger internationally operating corporations, entrepreneurs and their smaller companies also want to contribute to solving or mitigating the effects of the crisis. As OPC has the potential to increase problem solving and motivation (Avey et al., 2011), we theorize that solidarity and OCB are fostered by a company’s high level of psychological resources, as a need to promote the overall well-being increases (Hogan, 2020), which fosters such prosocial behaviors (Rodríguez et al., 2006). In fact, we find that higher levels of OPC increase the prosocial behaviors in SMEs during the COVID-19 pandemic, thus adding evidence that the positive psychological state of such companies increases desirable company actions in form of prosocial behaviors. We further argued that the investigated prosocial behaviors of SMEs enhance creative innovation of SMEs during the COVID-19 crisis and find proof for this assumption. OCB behaviors and solidarity actions show a positive impact on creative innovation, whereas the influence of OCB is stronger than the solidarity influence. Thus, we broaden the knowledge by showing SMEs in crises should leverage on those behaviors to increase their chances of survival.

Furthermore, we show that cooperation with stakeholders also increases when the SMEs can leverage on a high level of OPC. In order to mitigate the effects of crises, SMEs are lacking resources (Bin & Edwards, 2009; Fackler et al., 2013; Ogawa & Tanaka, 2013) and thus try to compensate for that by engaging in cooperation (Jones & Macpherson, 2006). To use their resources in an effective manner, research on this issues shows that for SMEs, it is crucial to understand their relations with stakeholders so that their goal directed energy can be enhanced (Battisti & Deakins, 2017). Having a high OPC fosters the motivation to counteract the crisis, and in consequence they leverage on different ways to overcome the situation and reach their goals (McKenny et al., 2013; Newman et al., 2014). As our results show, one of these ways is to engage in cooperation, as we find that creative innovation increases when SMEs engage in cooperation. Our findings contradicts the findings of Rosenbusch et al. (2011), which state that the yield of such cooperation has no effect on performance. They explain their findings in such a way that SMEs usually, due to their size, face a weaker negotiation power than their (bigger) cooperation partners and thus have to accept rather bad conditions. As the meta-analysis cannot account for the crisis context, we explain our findings in such a way that even though this is true in stable economic contexts, it differs in the COVID-19 pandemic, as the degree of novelty of those innovations is limited to the respective firm (Edison, 2013; Grimpe et al., 2017). We conclude that the positive effects of the cooperation, like information and knowledge gathering (Wall & Bellamy, 2019), mobilization of bricolage (Kuckertz et al., 2020) and joint efforts (Markman et al., 2019), weigh stronger.

In sum we add insights to entrepreneurship literature on the so far rather overlooked importance of positive psychological factors during crises and in our case OPC on the performance of SMEs, showing potential for further research. While we also agree with previous research showing that the context in which SMEs are embedded has a major impact on performance (Rosenbusch et al., 2011), we believe that OPC and other psychological variables may play a cross-contextual role determining performance during crises as well as in stable times. Therefore, we believe that further research in this direction is needed to help SMEs understand the potential entailed in OPC as a competitive advantage and what they can do in order to improve their resilience for future crises.

## Conclusion, limitations, and implications

Goal of our study was to investigate the factors that help SMEs to gain a better performance by leveraging on creative innovation during the COVID-19 pandemic. We propose that a company’s positive psychological state (OPC) plays a crucial role in this perspective, as it not only directly influences creative innovation and performance but also plays a decisive role in fostering prosocial behaviors (OCB and solidarity), as well as cooperation with stakeholders, which in turn influence small-scale creative innovation during a crisis. By that we follow the call of POB researchers to use a positive psychology perspective (James et al., 2011; Pearson & Clair, 1998) to enhance the understanding of SMEs performance in crises situations. We add to entrepreneurship literature by showing that OPC of SMEs positively impacts their performance during the COVID-19 pandemic and thus broadening the understanding of why some SMEs perform better during a crisis than others. Furthermore, we add to the crisis management literature by suggesting that positive psychological resources can be leveraged and thus should be considered to successfully navigate through crises. Last, as the COVID-19 crisis already endured over several months, we got the rare opportunity to conduct our study during a global crisis. Most research so far has been focused on either pre-crisis (preparation, causes) or post-crisis (aftermaths) situations (Williams et al., 2017).

Even though the recent COVID-19 pandemic offers great potential to study the behavior of companies in prolonged global crisis situations each crisis is different in its nature (James et al., 2011). In order to enhance and deepen our understanding of those different context, further research should validate our findings in different crises situations, as it is possible that in other crisis circumstances and different cultural backgrounds, prosocial behaviors as well as OPC operate in a different way, especially if the crisis is not classified as a grand challenge by the actors. Research in this direction could provide fruitful insights into the performance of SMEs during different crises and how those can prepare their organizations in advance by enhancing their OPC to better navigate through those times. Moreover, longitudinal research that can provide additional data from pre-crisis and post-crisis situation would enrich the discussion by shedding more light on the specific context in which each company is embedded. This would also allow for closer and multifaceted considerations into the nature of OPC, as it is assumed to change over time (Avey et al., 2010b; Peterson et al., 2011) and possibly decreases during a prolonged period of a crisis. Future studies could also increase our understanding of OPC by measuring the construct within a multitude of members of the respective organizations, as we measured OPC through self-assessment of the SME’s leaders.

Our study also offers several implications for research and practice. As OPC is considered a state like resource (Luthans et al., 2007) and thus has the potential to be enhanced by interventions (Dello Russo & Stoykova, 2015), our results indicate that such interventions during crises might be beneficial for the survival of the company. SMEs should place a higher focus on their psychological state, the positive attitude of their leaders and employees, and their resilience and hope, efficacy, and optimism to better cope with the effects of a crisis. Consequences would be a stronger prosocial behavior, cooperation, and a higher solidarity, which in return strengthens the innovative creations ultimately driving the performance of SMEs in times of crises.

## Appendix

**Table 7:** Controls SEM Model

Control Path	Path coefficients	T-values (*p* values)	Effect significant
Employees → Org. citizenship behavior	0.031	0.636 (0.525)	No
Employees → Cooperation	0.069	1.622 (0.105)	No
Employees → Solidarity	0.036	1.010 (0.313)	No
Employees → Creative innovation	− 0.011	0.342 (0.732)	No
Employees → Performance	0.015	0.313 (0.754)	No
Firm age → Org. citizenship behavior	− 0.112	2.226 (0.026)	Yes
Firm age → Cooperation	− 0.034	0.652 (0.515)	No
Firm age → Solidarity	− 0.011	0.218 (0.827)	No
Firm age → Creative innovation	− 0.077	1.958 (0.050)	Yes
Firm age → Performance	− 0.011	0.201 (0.841)	No
Industry — Manufacturing → Org. citizenship behavior	− 0.125	1.503 (0.133)	No
Industry — Manufacturing → Cooperation	− 0.126	1.450 (0.147)	No
Industry — Manufacturing → Solidarity	0.018	0.185 (0.853)	No
Industry — Manufacturing → Creative innovation	− 0.126	1.954 (0.051)	No
Industry — Manufacturing → Performance	− 0.028	0.370 (0.712)	No
Industry — Service → Org. citizenship behavior	0.101	1.248 (0.212)	No
Industry — Service → Cooperation	− 0.002	0.026 (0.979)	No
Industry — Service → Solidarity	0.079	0.823 (0.410)	No
Industry — Service → Creative innovation	− 0.007	0.114 (0.909)	No
Industry — Service → Performance	− 0.034	0.461 (0.645)	No
Female → Org. citizenship behavior	0.073	1.564 (0.118)	No
Female → Cooperation	0.067	1.256 (0.209)	No
Female → Solidarity	− 0.020	0.383 (0.701)	No
Female → Creative innovation	0.047	1.228 (0.219)	No
Female → Performance	− 0.078	1.841 (0.066)	No
Crisis-affected → Org. citizenship behavior	− 0.042	0.792 (0.429)	No
Crisis-affected → Cooperation	0.132	2.418 (0.016)	Yes
Crisis-affected → Solidarity	− 0.056	0.987 (0.324)	No
Crisis-affected → Creative innovation	0.061	1.406 (0.160)	No
Crisis-affected → Performance	− 0.450	10.549 (0.000)	Yes

## Footnotes

1 Research Edition Translation of the Psychological Capital Questionnaire—Self Form performed by Ann-Christin Grözinger, Dr. Sven Wolff and Prof. Dr. Petra Moog. Translated into German, altered and reproduced by special permission of the publisher, Mind Garden, Inc., www.mindgarden.com, from the Psychological Capital Questionnaire—Self Form by Fred L. Luthans, Bruce J. Avolio & James B. Avey. Copyright © 2007 Psychological Capital (PsyCap) Questionnaire (PCQ) Fred L. Luthans, Bruce J. Avolio & James B. Avey. All rights reserved in all medium. Further reproduction is prohibited without the Publisher's written consent. Altered with permission of the publisher.

## References

[CR1] Acs, Z. J., & Audretsch, D. B. (1988). Innovation in large and small firms: An empirical analysis. *The American Economic Review,**78*(4), 678–690.

[CR2] Acs, Z. J., & Phillips, R. J. (2002). Entrepreneurship and philanthropy in American capitalism. *Small Business Economics,**19*(3), 189–204. 10.1023/A:1019635015318

[CR3] Alrich, H., & Auster, E. (1986). Even dwarfs started small: Liabilities of age and size and their strategic implications. *Research in Organizational Behavior,**8*, 165–198.

[CR4] Amabile, T. M. (1988). A model of creativity and innovation in organizations. *Research in Organizational Behavior,**10*, 123–167.

[CR5] Amabile, T. M., & Pratt, M. G. (2016). The dynamic componential model of creativity and innovation in organizations: Making progress, making meaning. *Research in Organizational Behavior,**36*, 157–183. 10.1016/j.riob.2016.10.001

[CR6] Anderson, R. C., & Reeb, D. M. (2003). Founding-family ownership and firm performance: Evidence from the S&P 500. *The Journal of Finance,**58*(3), 1301–1328. 10.1111/1540-6261.00567

[CR7] Andries, P., & Czarnitzki, D. (2014). Small firm innovation performance and employee involvement. *Small Business Economics,**43*(1), 21–38. 10.1007/s11187-014-9577-1

[CR8] Armstrong, J. S., & Overton, T. S. (1977). Estimating nonresponse bias in mail surveys. *Journal of Marketing Research,**14*(3), 396–402. 10.2307/3150783

[CR9] Arregle, J.-L., Hitt, M. A., Sirmon, D. G., & Very, P. (2007). The development of organizational social capital: Attributes of Family Firms. *Journal of Management Studies,**44*(1), 73–95. 10.1111/j.1467-6486.2007.00665.x

[CR10] Avey, J. B., Wernsing, T. S., & Luthans, F. (2008). Can positive employees help positive organizational change? Impact of psychological capital and emotions on relevant attitudes and behaviors. *The Journal of Applied Behavioral Science,**44*(1), 48–70. 10.1177/0021886307311470

[CR11] Avey, J. B., Luthans, F., Smith, R. M., & Palmer, N. F. (2010a). Impact of positive psychological capital on employee well-being over time. *Journal of Occupational Health Psychology,**15*(1), 17–28. 10.1037/a0016998 20063956 10.1037/a0016998

[CR12] Avey, J. B., Nimnicht, J. L., & Graber Pigeon, N. (2010b). Two field studies examining the association between positive psychological capital and employee performance. *Leadership & Organization Development Journal,**31*(5), 384–401. 10.1108/01437731011056425

[CR13] Avey, J. B., Reichard, R. J., Luthans, F., & Mhatre, K. H. (2011). Meta-analysis of the impact of positive psychological capital on employee attitudes, behaviors, and performance. *Human Resource Development Quarterly,**22*(2), 127–152. 10.1002/hrdq.20070

[CR14] Avolio, B. J., & Gardner, W. L. (2005). Authentic leadership development: Getting to the root of positive forms of leadership. *The Leadership Quarterly, 16*(3), 315–338. 10.1016/j.leaqua.2005.03.001

[CR15] Backman, M., & Palmberg, J. (2015). Contextualizing small family firms: How does the urban–rural context affect firm employment growth? *Journal of Family Business Strategy,**6*(4), 247–258. 10.1016/j.jfbs.2015.10.003

[CR16] Bacq, S., Geoghegan, W., Josefy, M., Stevenson, R., & Williams, T. A. (2020). The COVID-19 virtual idea Blitz: Marshaling social entrepreneurship to rapidly respond to urgent grand challenges. *Business Horizons, 63*(6), 705–723. 10.1016/j.bushor.2020.05.002 10.1016/j.bushor.2020.05.002 PMC7214311 32398883

[CR17] Bandura, A. (1997). *Self efficacy: The exercise of control*. Worth.

[CR18] Bandura, A. (2012). On the functional properties of perceived self-efficacy revisited. *Journal of Management,**38*(1), 9–44. 10.1177/0149206311410606

[CR19] Barney, J. (1991). Firm resources and sustained competitive advantage. *Journal of Management,**17*(1), 99–120. 10.1177/014920639101700108

[CR20] Barrick, M. R., & Mount, M. K. (1991). The Big Five personality dimensions and job performance: A meta-analysis. *Personnel Psychology,**44*(1), 1–26. 10.1111/j.1744-6570.1991.tb00688.x

[CR21] Bartik, A. W., Bertrand, M., Cullen, Z., Glaeser, E. L., Luca, M., & Stanton, C. (2020). The impact of COVID-19 on small business outcomes and expectations. *Proceedings of the National Academy of Sciences,**117*(30), 17656–17666. 10.1073/pnas.2006991117 10.1073/pnas.2006991117 PMC7395529 32651281

[CR22] Battisti, M., & Deakins, D. (2017). The relationship between dynamic capabilities, the firm’s resource base and performance in a post-disaster environment. *International Small Business Journal,**35*(1), 78–98. 10.1177/0266242615611471

[CR23] Belderbos, R., Carree, M., & Lokshin, B. (2006). Complementarity in R&D cooperation strategies. *Review of Industrial Organization,**28*(4), 401–426. 10.1007/s11151-006-9102-z

[CR24] Beliaeva, T., Shirokova, G., Wales, W., & Gafforova, E. (2020). Benefiting from economic crisis? Strategic orientation effects, trade-offs, and configurations with resource availability on SME performance. *International Entrepreneurship and Management Journal,**16*(1), 165–194. 10.1007/s11365-018-0499-2

[CR25] Bin, O., & Edwards, B. (2009). Social capital and business giving to charity following a natural disaster: An empirical assessment. *The Journal of Socio-Economics,**38*(4), 601–607. 10.1016/j.socec.2009.02.010

[CR26] Bongini, P., Ferrando, A., Rossi, E., & Rossolini, M. (2021). SME access to market-based finance across Eurozone countries. *Small Business Economics, 56*(4), 1667–1697. 10.1007/s11187-019-00285-z

[CR27] Broad, J., & Luthans, F. (2017). Leading and developing health and safety through collective psychological capital. In E. K. Kelloway, K. Nielsen, & J. K. Dimoff (Eds.), *Leading to occupational health and safety: How leadership behaviours impact organizational safety and well-being.* (pp. 225–280). Wiley.

[CR28] Buerau van Dijk. (2020, Juni 1). *Buerau van Dijk—A Moody’s Analytics company*. Buerau van Dijk - A Moody’s Analytics Company. www.bvdinfo.com.

[CR29] Chan, D. (1998). Functional relations among constructs in the same content domain at different levels of analysis: A typology of composition models. *Journal of Applied Psychology,**83*(2), 234–246. 10.1037/0021-9010.83.2.234

[CR30] Chen, G., Mathieu, J. E., & Bliese, P. D. (2004). A Framework for conducting multi-level construct validation. In F. J. Yammarino & F. Dansereau (Eds.), *Multi-level Issues in Organizational Behavior and Processes.* (pp. 273–303). Emerald Group Publishing Limited. 10.1016/S1475-9144(04)03013-9

[CR31] Chin, W. W. (1998). The partial least squares approach to structural equation modeling. In G. A. Marcoulides (Ed.), *Modern Methods for Business Research.* (pp. 295–358). Lawrence Erlbaum.

[CR32] Clapp-Smith, R., Vogelgesang, G. R., & Avey, J. B. (2009). Authentic leadership and positive psychological capital: The mediating role of trust at the group level of analysis. *Journal of Leadership & Organizational Studies,**15*(3), 227–240. 10.1177/1548051808326596

[CR33] Collett, N., Pandit, N. R., & Saarikko, J. (2014). Success and failure in turnaround attempts. An analysis of SMEs within the Finnish Restructuring of Enterprises Act. *Entrepreneurship & Regional Development,**26*(1–2), 123–141. 10.1080/08985626.2013.870236

[CR34] Cowling, M., Liu, W., & Zhang, N. (2018). Did firm age, experience, and access to finance count? SME performance after the global financial crisis. *Journal of Evolutionary Economics,**28*(1), 77–100. 10.1007/s00191-017-0502-z

[CR35] Crook, T. R., Ketchen, D. J., Combs, J. G., & Todd, S. Y. (2008). Strategic resources and performance: A meta-analysis. *Strategic Management Journal,**29*(11), 1141–1154. 10.1002/smj.703

[CR36] Cucculelli, M., & Peruzzi, V. (2020). Post-crisis firm survival, business model changes, and learning: Evidence from the Italian manufacturing industry. *Small Business Economics,**54*(2), 459–474. 10.1007/s11187-018-0044-2

[CR37] Davidsson, P., Achtenhagen, L., & Naldi, L. (2010). Small firm growth. *Foundations and Trends in Entrepreneurship,**6*(2), 69–166. 10.1561/0300000029

[CR38] Dawkins, S., Martin, A., Scott, J., & Sanderson, K. (2015). Advancing conceptualization and measurement of psychological capital as a collective construct. *Human Relations,**68*(6), 925–949. 10.1177/0018726714549645

[CR39] Dawkins, S., Martin, A., Scott, J., Sanderson, K., & Schüz, B. (2018). A cross-level model of team-level psychological capital (PsyCap) and individual- and team-level outcomes. *Journal of Management & Organization*, 1–20.10.1017/jmo.2018.27.

[CR40] Dehlen, T., Zellweger, T., Kammerlander, N., & Halter, F. (2014). The role of information asymmetry in the choice of entrepreneurial exit routes. *Journal of Business Venturing,**29*(2), 193–209. 10.1016/j.jbusvent.2012.10.001

[CR41] Delery, J. E., & Roumpi, D. (2017). Strategic human resource management, human capital and competitive advantage: Is the field going in circles? *Human Resource Management Journal,**27*(1), 1–21. 10.1111/1748-8583.12137

[CR42] Dello Russo, S., & Stoykova, P. (2015). Psychological capital intervention (PCI): A replication and extension. *Human Resource Development Quarterly,**26*(3), 329–347. 10.1002/hrdq.21212

[CR43] Dess, G. G., & Robinson, R. B. (1984). Measuring organizational performance in the absence of objective measures: The case of the privately-held firm and conglomerate business unit. *Strategic Management Journal,**5*(3), 265–273. 10.1002/smj.4250050306

[CR44] Dewald, J., & Bowen, F. (2010). Storm clouds and silver linings: Responding to disruptive innovations through cognitive resilience. *Entrepreneurship Theory and Practice,**34*(1), 197–218. 10.1111/j.1540-6520.2009.00312.x

[CR45] Doern, R., Williams, N., & Vorley, T. (2019). Special issue on entrepreneurship and crises: Business as usual? An introduction and review of the literature. *Entrepreneurship & Regional Development,**31*(5–6), 400–412. 10.1080/08985626.2018.1541590

[CR46] Eddleston, K. A., Kellermanns, F. W., & Sarathy, R. (2007). Resource configuration in family firms: Linking resources, strategic planning and technological opportunities to performance. *Journal of Management Studies,**45*(1), 26–50. 10.1111/j.1467-6486.2007.00717.x

[CR47] Edison, H., Bin Ali, N., & Torkar, R. (2013). Towards innovation measurement in the software industry. *Journal of Systems and Software,**86*(5), 1390–1407. 10.1016/j.jss.2013.01.013

[CR48] European Commission. (2011). *Commission Regulation (EU) No 715/2010 of 10 August 2010 amending Council Regulation (EC) No 2223/96 as regards adaptations following the revision of the statistical classification of economic activities NACE Revision 2 and the statistical classification of products by activity (CPA) in national accounts*. 21.

[CR49] Fackler, D., Schnabel, C., & Wagner, J. (2013). Establishment exits in Germany: The role of size and age. *Small Business Economics,**41*(3), 683–700. 10.1007/s11187-012-9450-z

[CR50] Fornell, C., & Larcker, D. F. (1981). Evaluating structural equation models with unobservable variables and measurement error. *Journal of Marketing Research,**18*(1), 39–50. 10.1177/002224378101800104

[CR51] Fox, S., & Spector, P. E. (2011). *Organizational citizenship behavior checklist (OCB-C)*. CWB-C. http://shell.cas.usf.edu/~pspector/scales/ocbcpage.html.

[CR52] Fuller, C. M., Simmering, M. J., Atinc, G., Atinc, Y., & Babin, B. J. (2016). Common methods variance detection in business research. *Journal of Business Research,**69*(8), 3192–3198. 10.1016/j.jbusres.2015.12.008

[CR53] Gable, S. L., & Haidt, J. (2005). What (and why) is positive psychology? *Review of General Psychology,**9*(2), 103–110. 10.1037/1089-2680.9.2.103

[CR54] George, G., Lakhani, K. R., & Puranam, P. (2020). What has changed? The impact of Covid pandemic on the technology and innovation management research agenda. *Journal of Management Studies,**57*(8), 1754–1758. 10.1111/joms.12634

[CR55] Glass, R. J., Glass, L. M., Beyeler, W. E., & Min, H. J. (2006). Targeted social distancing design for pandemic influenza. *Emerging Infectious Diseases,**12*(11), 11. 10.3201/eid1211.060255 PMC3372334 17283616

[CR56] Global Data PLC. (2020). *Coronavirus (COVID-19) Executive Briefing*. Global Data.

[CR57] Gooty, J., Gavin, M., Johnson, P. D., Frazier, M. L., & Snow, D. B. (2009). In the eyes of the beholder: Transformational leadership, positive psychological capital, and performance. *Journal of Leadership & Organizational Studies, 15*(4), 353–367. 10.1177/1548051809332021

[CR58] Grimpe, C., Sofka, W., Bhargava, M., & Chatterjee, R. (2017). R&D, marketing innovation, and new product performance: A mixed methods study. *Journal of Product Innovation Management,**34*(3), 360–383. 10.1111/jpim.12366

[CR59] Hair, J. F., Hult, G. T. M., Ringle, C. M., & Sarstedt, M. (2016). *A primer on partial least squares structural equations modeling (PLS-SEM)*. (2nd ed.). SAGE.

[CR60] Hair, J. F., Ringle, C. M., & Sarstedt, M. (2011). PLS-SEM: Indeed a silver bullet. *Journal of Marketing Theory and Practice,**19*(2), 139–152. 10.2753/MTP1069-6679190202

[CR61] Hair, J. F., Risher, J. J., Sarstedt, M., & Ringle, C. M. (2019). When to use and how to report the results of PLS-SEM. *European Business Review,**31*(1), 2–24. 10.1108/EBR-11-2018-0203

[CR62] Hansen, B., & Hamilton, R. T. (2011). Factors distinguishing small firm growers and non-growers. *International Small Business Journal: Researching Entrepreneurship,**29*(3), 278–294. 10.1177/0266242610381846

[CR63] Hansen, D. J., Lumpkin, G. T., & Hills, G. E. (2011). A multidimensional examination of a creativity-based opportunity recognition model. *International Journal of Entrepreneurial Behavior & Research,**17*(5), 515–533. 10.1108/13552551111158835

[CR64] He, H., & Harris, L. (2020). The impact of Covid-19 pandemic on corporate social responsibility and marketing philosophy. *Journal of Business Research,**116*, 176–182. 10.1016/j.jbusres.2020.05.030 32457556 10.1016/j.jbusres.2020.05.030 PMC7241379

[CR65] Helton, W. S., & Head, J. (2012). Earthquakes on the mind: Implications of disasters for human performance. *Human Factors: the Journal of the Human Factors and Ergonomics Society,**54*(2), 189–194. 10.1177/0018720811430503 10.1177/0018720811430503 22624286

[CR66] Herbane, B. (2010). Small business research: Time for a crisis-based view. *International Small Business Journal: Researching Entrepreneurship,**28*(1), 43–64. 10.1177/0266242609350804

[CR67] Heunks, F. J. (1998). Innovation, creativity and success. *Small Business Economics,**10*(3), 263–272. 10.1023/A:1007968217565

[CR68] Hitt, M. A., Bierman, L., Shimizu, K., & Kochhar, R. (2001). Direct and moderating effects of human capital on strategy and performance in professional service firms: A resource-based perspective. *Academy of Management Journal,**44*(1), 13–28. 10.5465/3069334

[CR69] Hmieleski, K. M., Carr, J. C., & Baron, R. A. (2015). Integrating discovery and creation perspectives of entrepreneurial action: The relative roles of founding CEO human capital, social capital, and psychological capital in contexts of risk versus uncertainty: Discovery and creation perspectives of entrepreneurial action. *Strategic Entrepreneurship Journal,**9*(4), 289–312. 10.1002/sej.1208

[CR70] Hobfoll, S. E. (2002). Social and psychological resources and adaptation. *Review of General Psychology,**6*(4), 307–324. 10.1037/1089-2680.6.4.307

[CR71] Hogan, M. J. (2020). Collaborative positive psychology: Solidarity, meaning, resilience, wellbeing, and virtue in a time of crisis. *International Review of Psychiatry,**32*(7–8), 698–712. 10.1080/09540261.2020.1778647 33427525 10.1080/09540261.2020.1778647

[CR72] Howard‐Grenville, J. (2021). Grand challenges, Covid‐19 and the future of organizational scholarship. *Journal of Management Studies, 58*(1), 254–258. 10.1111/joms.12647

[CR73] Hu, L., & Bentler, P. M. (1999). Cutoff criteria for fit indexes in covariance structure analysis: Conventional criteria versus new alternatives. *Structural Equation Modeling: A Multidisciplinary Journal,**6*(1), 1–55. 10.1080/10705519909540118

[CR74] Huang, L., & Luthans, F. (2015). Toward better understanding of the learning goal orientation-creativity relationship: The role of positive psychological capital. *Applied Psychology,**64*(2), 444–472. 10.1111/apps.12028

[CR75] Ibn-Mohammed, T., Mustapha, K. B., Godsell, J., Adamu, Z., Babatunde, K. A., Akintade, D. D., Acquaye, A., Fujii, H., Ndiaye, M. M., Yamoah, F. A., & Koh, S. C. L. (2021). A critical analysis of the impacts of COVID-19 on the global economy and ecosystems and opportunities for circular economy strategies. *Resources, Conservation and Recycling,**164*, 1–22. 10.1016/j.resconrec.2020.105169 10.1016/j.resconrec.2020.105169 PMC7505605 32982059

[CR76] James, E. H., Wooten, L. P., & Dushek, K. (2011). Crisis management: Informing a new leadership research agenda. *Academy of Management Annals,**5*(1), 455–493. 10.5465/19416520.2011.589594

[CR77] Jonas, K. J. (2012). Prosocial behavior in the context of crisis. In K. J. Jonas & T. A. Morton (Eds.), *Restoring Civil Societies.* (pp. 57–77). Wiley. 10.1002/9781118347683.ch4

[CR78] Jones, O., & Macpherson, A. (2006). Inter-organizational learning and strategic renewal in SMEs. *Long Range Planning,**39*(2), 155–175. 10.1016/j.lrp.2005.02.012

[CR79] Karlsson, J. (2020). Firm size and growth barriers: A data-driven approach. *Small Business Economics*. 10.1007/s11187-020-00350-y

[CR80] Kiefer, K., Heileman, M., & Pett, T. L. (2020). Does gender still matter? An examination of small business performance. *Small Business Economics*. 10.1007/s11187-020-00403-2

[CR81] Kottika, E., Özsomer, A., Rydén, P., Theodorakis, I. G., Kaminakis, K., Kottikas, K. G., & Stathakopoulos, V. (2020). We survived this! What managers could learn from SMEs who successfully navigated the Greek economic crisis. *Industrial Marketing Management,**88*, 352–365. 10.1016/j.indmarman.2020.05.021

[CR82] Kozlowski, S., & Klein, K. (2000). A multilevel approach to theory and research in organizations: Contextual, temporal, and emergent processes. In K. Klein & S. Kozlowski (Eds.), *Multilevel theory, research and methods in organizations: Foundations, extensions, and new directions.* (Corr 3, pp. 3–90). Wiley.

[CR83] Kuckertz, A., Brändle, L., Gaudig, A., Hinderer, S., Morales Reyes, C. A., Prochotta, A., Steinbrink, K. M., & Berger, E. S. C. (2020). Startups in times of crisis – A rapid response to the COVID-19 pandemic. *Journal of Business Venturing Insights,**13*, 1–13. 10.1016/j.jbvi.2020.e00169

[CR84] Kusunoki, K., Nonaka, I., & Nagata, A. (1998). Organizational capabilities in product development of Japanese Firms: A conceptual framework and empirical findings. *Organization Science,**9*(6), 699–718. 10.1287/orsc.9.6.699

[CR85] Lam, C. F., Wan, W. H., & Roussin, C. J. (2016). Going the extra mile and feeling energized: An enrichment perspective of organizational citizenship behaviors. *Journal of Applied Psychology,**101*(3), 379–391. 10.1037/apl0000071 26595756 10.1037/apl0000071

[CR86] Larson, M., & Luthans, F. (2006). Potential added value of psychological capital in predicting work attitudes. *Journal of Leadership and Organizational Studies,**13*(2), 75–92. 10.1177/10717919070130020601

[CR87] Latham, S. (2009). Contrasting strategic response to economic recession in start-up versus established software firms. *Journal of Small Business Management,**47*(2), 180–201. 10.1111/j.1540-627X.2009.00267.x

[CR88] Lengnick-Hall, C. A., & Beck, T. E. (2005). Adaptive fit versus robust transformation: How organizations respond to environmental change. *Journal of Management,**31*(5), 738–757. 10.1177/0149206305279367

[CR89] Li, S., & Tallman, S. (2011). MNC strategies, exogenous shocks, and performance outcomes. *Strategic Management Journal,**32*(10), 1119–1127. 10.1002/smj.918

[CR90] Lins, K. V., Servaes, H., & Tamayo, A. (2017). Social capital, trust, and firm performance: The value of corporate social responsibility during the financial crisis: social capital, trust, and firm performance. *The Journal of Finance,**72*(4), 1785–1824. 10.1111/jofi.12505

[CR91] Love, L. G., Priem, R. L., & Lumpkin, G. T. (2002). Explicitly articulated strategy and firm performance under alternative levels of centralization. *Journal of Management,**28*(5), 611–627. 10.1177/014920630202800503

[CR92] Lumpkin, G. T., & Lichtenstein, B. B. (2005). The role of organizational learning in the opportunity–recognition process. *Entrepreneurship Theory and Practice,**29*(4), 451–472. 10.1111/j.1540-6520.2005.00093.x

[CR93] Luthans, F. (2002a). Positive organizational behavior: Developing and managing psychological strengths. *Academy of Management Perspectives,**16*(1), 57–72. 10.5465/ame.2002.6640181

[CR94] Luthans, F. (2002b). The need for and meaning of positive organizational behavior. *Journal of Organizational Behavior,**23*(6), 695–706. 10.1002/job.165

[CR95] Luthans, F., & Youssef, C. M. (2004). Human, social, and now positive psychological capital management: Investing in people for competitive advantage. *Organizational Dynamics,**33*(2), 143–160. 10.1016/j.orgdyn.2004.01.003

[CR96] Luthans, F., & Youssef-Morgan, C. M. (2017). Psychological capital: an evidence-based positive approach. *Annual Review of Organizational Psychology and Organizational Behavior,**4*(1), 339–366. 10.1146/annurev-orgpsych-032516-113324

[CR97] Luthans, F., Avolio, B. J., Walumbwa, F. O., & Li, W. (2005). The psychological capital of Chinese workers: Exploring the relationship with performance. *Management and Organization Review,**1*(2), 249–271. 10.1111/j.1740-8784.2005.00011.x

[CR98] Luthans, F., Vogelgesang, G. R., & Lester, P. B. (2006). Developing the psychological capital of resiliency. *Human Resource Development Review,**5*(1), 25–44. 10.1177/1534484305285335

[CR99] Luthans, F., Avolio, B. J., Avey, J. B., & Norman, S. M. (2007). Positive psychological capital: Measurement and relationship with performance and satisfaction. *Personnel Psychology,**60*(3), 541–572. 10.1111/j.1744-6570.2007.00083.x

[CR100] Luthans, F., Avey, J. B., Clapp-Smith, R., & Li, W. (2008). More evidence on the value of Chinese workers’ psychological capital: A potentially unlimited competitive resource? *The International Journal of Human Resource Management,**19*(5), 818–827. 10.1080/09585190801991194

[CR101] Luthans, F., Avey, J. B., Avolio, B. J., & Peterson, S. J. (2010). The development and resulting performance impact of positive psychological capital. *Human Resource Development Quarterly,**21*(1), 41–67. 10.1002/hrdq.20034

[CR102] Luthans, F., Youssef, C. M., & Rawski, S. L. (2011). A tale of two paradigms: The impact of psychological capital and reinforcing feedback on problem solving and innovation. *Journal of Organizational Behavior Management,**31*(4), 333–350. 10.1080/01608061.2011.619421

[CR103] Luthans, F., Youssef-Morgan, C. M., & Avolio, B. J. (2015). *Psychological capital and beyond* (Har/Psc). OUP USA. 10.1002/smi.2623 26250352

[CR104] Markman, G. D., Waldron, T. L., Gianiodis, P. T., & Espina, M. I. (2019). E Pluribus Unum: Impact entrepreneurship as a solution to grand challenges. *Academy of Management Perspectives,**33*(4), 371–382. 10.5465/amp.2019.0130

[CR105] Masten, A. S. (2001). Resilience processes in development. *American Psychologist,**56*(3), 227–238. 10.1007/0-306-48572-9_2 11315249 10.1037//0003-066x.56.3.227

[CR106] Masten, A. S., & Reed, M.-G.J. (2002). Resilience in development. In S. J. Lopez & C. R. Snyder (Eds.), *Oxford Handbook of Positive Psychology. *Oxford University Press.

[CR107] Mathe, K., Scott-Halsell, S., Kim, S., & Krawczyk, M. (2017). Psychological capital in the Quick Service Restaurant Industry: A study of unit-level performance. *Journal of Hospitality & Tourism Research,**41*(7), 823–845. 10.1177/1096348014550923

[CR108] Mayr, S., Mitter, C., & Aichmayr, A. (2017). Corporate crisis and sustainable reorganization: Evidence from Bankrupt Austrian SMEs. *Journal of Small Business Management,**55*(1), 108–127. 10.1111/jsbm.12248

[CR109] McKenny, A. F., Short, J. C., & Payne, G. T. (2013). Using computer-aided text analysis to elevate constructs: An illustration using psychological capital. *Organizational Research Methods,**16*(1), 152–184. 10.1177/1094428112459910

[CR110] Memili, E., Welsh, D. H. B., & Luthans, F. (2013). Going beyond research on goal setting: A proposed role for organizational psychological capital of family firms. *Entrepreneurship Theory and Practice,**37*(6), 1289–1296. 10.1111/etap.12066

[CR111] Milosevic, I., Bass, A. E., & Milosevic, D. (2017). Leveraging positive psychological capital (PsyCap) in crisis: A multiphase framework. *Organization Management Journal,**14*(3), 127–146. 10.1080/15416518.2017.1353898

[CR112] Naldi, L., Nordqvist, M., Sjöberg, K., & Wiklund, J. (2007). Entrepreneurial orientation, risk taking, and performance in family firms. *Family Business Review,**20*(1), 33–47. 10.1111/j.1741-6248.2007.00082.x

[CR113] Newman, A., Ucbasaran, D., Zhu, F., & Hirst, G. (2014). Psychological capital: A review and synthesis. *Journal of Organizational Behavior,**35*(1), 120–138. 10.1002/job.1916

[CR114] Nooteboom, B. (1994). Innovation and diffusion in small firms: Theory and evidence. *Small Business Economics,**6*(5), 327–347. 10.1007/BF01065137

[CR115] Norman, S. M., Avey, J. B., Nimnicht, J. L., & Graber Pigeon, N. (2010). The interactive effects of psychological capital and organizational identity on employee organizational citizenship and deviance behaviors. *Journal of Leadership & Organizational Studies,**17*(4), 380–391. 10.1177/1548051809353764

[CR116] Ogawa, K., & Tanaka, T. (2013). The global financial crisis and small- and medium-sized enterprises in Japan: How did they cope with the crisis? *Small Business Economics,**41*(2), 401–417. 10.1007/s11187-012-9434-z

[CR117] Organ, D. W. (1997). Organizational citizenship behavior: It’s construct clean-up time. *Human Performance,**10*(2), 85–97. 10.1207/s15327043hup1002_2

[CR118] Organ, D. W. (2018). Organizational citizenship behavior: Recent trends and developments. *Annual Review of Organizational Psychology and Organizational Behavior,**5*(1), 295–306. 10.1146/annurev-orgpsych-032117-104536

[CR119] Pal, R., Torstensson, H., & Mattila, H. (2014). Antecedents of organizational resilience in economic crises—An empirical study of Swedish textile and clothing SMEs. *International Journal of Production Economics,**147*, 410–428. 10.1016/j.ijpe.2013.02.031

[CR120] Pearson, C. M., & Clair, J. A. (1998). Reframing crisis management. *The Academy of Management Review,**23*(1), 59–76. 10.2307/259099

[CR121] Penrose, J. M. (2000). The role of perception in crisis planning. *Public Relations Review,**26*(2), 155–171. 10.1016/S0363-8111(00)00038-2

[CR122] Pérez, A., & Rodríguez del Bosque, I. (2013). Measuring CSR image: Three studies to develop and to validate a reliable measurement tool. *Journal of Business Ethics,**118*(2), 265–286. 10.1007/s10551-012-1588-8

[CR123] Peterson, S. J., & Zhang, Z. (2011). Examining the relationships between top management team psychological characteristics, transformational leadership, and business unit performance. In M. A. Carpenter (Ed.), *The Handbook of Research on Top Management Teams.* (pp. 127–149). Edward Elgar Publishing Ltd.

[CR124] Peterson, S. J., Luthans, F., Avolio, B. J., Walumbwa, F., & Zhang, Z. (2011). Psychological capital and employee performance: A latent growth modeling approach. *Personnel Psychology,**64*(2), 427–450. 10.1111/j.1744-6570.2011.01215.x

[CR125] Podsakoff, P. M., MacKenzie, S. B., Moorman, R. H., & Fetter, R. (1990). Transformational leader behaviors and their effects on followers’ trust in leader, satisfaction, and organizational citizenship behaviors. *The Leadership Quarterly,**1*(2), 107–142. 10.1016/1048-9843(90)90009-7

[CR126] Podsakoff, P. M., MacKenzie, S. B., Lee, J.-Y., & Podsakoff, N. P. (2003). Common method biases in behavioral research: A critical review of the literature and recommended remedies. *Journal of Applied Psychology,**88*(5), 879–903. 10.1037/0021-9010.88.5.879 14516251 10.1037/0021-9010.88.5.879

[CR127] Porter, M. E. (1980). *Competitive strategy: Techniques for analyzing industries and competitors*. New York: Free Press.

[CR128] Raja, U., Azeem, M. U., Haq, I. U., & Naseer, S. (2020). Perceived threat of terrorism and employee outcomes: The moderating role of negative affectivity and psychological capital. *Journal of Business Research,**110*, 316–326. 10.1016/j.jbusres.2020.01.026

[CR129] Rego, A., Sousa, F., Marques, C., Cunha, M. P., & e. . (2012). Authentic leadership promoting employees’ psychological capital and creativity. *Journal of Business Research,**65*(3), 429–437. 10.1016/j.jbusres.2011.10.003

[CR130] Robert Koch Institut. (2020). *Coronavirus disease 2019 (COVID-19) daily situation report of the Robert Koch Institute* (S. 8). https://www.rki.de/DE/Content/InfAZ/N/Neuartiges_Coronavirus/Situationsberichte/2020-07-31-en.pdf?__blob=publicationFile. Accessed 10 Jan 2021.

[CR131] Rodríguez, H., Trainor, J., & Quarantelli, E. L. (2006). Rising to the challenges of a catastrophe: The emergent and prosocial behavior following Hurricane Katrina. *The ANNALS of the American Academy of Political and Social Science,**604*(1), 82–101. 10.1177/0002716205284677

[CR132] Rosenbusch, N., Brinckmann, J., & Bausch, A. (2011). Is innovation always beneficial? A meta-analysis of the relationship between innovation and performance in SMEs. *Journal of Business Venturing,**26*(4), 441–457. 10.1016/j.jbusvent.2009.12.002

[CR133] Rosso, B. D., Dekas, K. H., & Wrzesniewski, A. (2010). On the meaning of work: A theoretical integration and review. *Research in Organizational Behavior,**30*, 91–127. 10.1016/j.riob.2010.09.001

[CR134] Runyan, R. C. (2006). Small business in the face of crisis: Identifying barriers to recovery from a natural disaster1. *Journal of Contingencies and Crisis Management,**14*(1), 12–26. 10.1111/j.1468-5973.2006.00477.x

[CR135] Salancik, G. R., & Pfeffer, J. (1978). A social information processing approach to job attitudes and task design. *Administrative Science Quarterly, 23*(2), 224. 10.2307/2392563 10307892

[CR136] Scheier, M. F., Carver, C. S., & Bridges, M. W. (2001). Optimism, pessimism, and psychological well-being. In E. C. Chang (Ed.), *Optimism & pessimism: Implications for theory, research, and practice.* (pp. 189–216). American Psychological Association.

[CR137] Schumpeter, J. A. (1934). *The Theory of economic development*. Harvard University Press.

[CR138] Shan, W. (1990). An empirical analysis of organizational strategies by entrepreneurial high-technology firms. *Strategic Management Journal,**11*, 129–139. 10.1002/smj.4250110205

[CR139] Sirmon, D. G., & Hitt, M. A. (2003). Resources, management, and wealth creation in family firms. *Entrepreneurship Theory and Practice,**23*(2), 25–62. 10.1111/1540-8520.t01-1-00013

[CR140] Smallbone, D., Leig, R., & North, D. (1995). The characteristics and strategies of high growth SMEs. *International Journal of Entrepreneurial Behavior & Research,**1*(3), 44–62. 10.1108/13552559510100657

[CR141] Smallbone, D., Deakins, D., Battisti, M., & Kitching, J. (2012). Small business responses to a major economic downturn: Empirical perspectives from New Zealand and the United Kingdom. *International Small Business Journal: Researching Entrepreneurship,**30*(7), 754–777. 10.1177/0266242612448077

[CR142] Smolka, K. M., Verheul, I., Burmeister-Lamp, K., & Heugens, P. P. M. A. R. (2016). Get it together! Synergistic effects of causal and effectual decision-making logics on venture performance. *Entrepreneurship Theory and Practice,**42*(4), 1–34. 10.1111/etap.12266

[CR143] Snyder, C. R., Harris, C., Anderson, J. R., Holleran, S. A., Irving, L. M., & Sigmon, S. X. (1991). The will and the ways: Development and validation of an individual-differences measure of hope. *Journal of Personality and Social Psychology,**60*(4), 570–585. 10.1037//0022-3514.60.4.570 2037968 10.1037//0022-3514.60.4.570

[CR144] Snyder, C. R., Sympson, S. C., Ybasco, F. C., Borders, T. F., Babyak, M. A., & Higgins, R. L. (1996). Development and validation of the state hope scale. *Journal of Personality and Social Psychology,**70*(2), 321–335. 10.1037/0022-3514.70.2.321 8636885 10.1037//0022-3514.70.2.321

[CR145] Stajkovic, A. D., & Luthans, F. (1998). Self-efficacy and work-related performance: A meta-analysis. *Psychological Bulletin,**124*(2), 240–261. 10.1037/0033-2909.124.2.240

[CR146] Staw, B. M. (1990). An evolutionary approach to creativity and innovation. In M. A. West & J. L. Farr (Eds.), *Innovation and creativity at work: Psychological and organizational strategies.* (pp. 287–308). Wiley.

[CR147] Sullins, E. S. (1991). Emotional contagion revisited: Effects of social comparison and expressive style on mood convergence. *Personality and Social Psychology Bulletin, 17*(2), 166–174. 10.1177/014616729101700208

[CR148] Sweetman, D., Luthans, F., Avey, J. B., & Luthans, B. C. (2011). Relationship between positive psychological capital and creative performance. *Canadian Journal of Administrative Sciences / Revue Canadienne Des Sciences De L’administration,**28*(1), 4–13. 10.1002/cjas.175

[CR149] Tyler, B. B., & Steensma, H. K. (1998). The effects of executives’ experiences and perceptions on their assessment of potential technological alliances. *Strategic Management Journal,**19*(10), 939–965. 10.1002/(SICI)1097-0266(199810)19:10%3c939::AID-SMJ978%3e3.0.CO;2-Z

[CR150] Vargo, J., & Seville, E. (2011). Crisis strategic planning for SMEs: Finding the silver lining. *International Journal of Production Research,**49*(18), 5619–5635. 10.1080/00207543.2011.563902

[CR151] Vossen, R. W. (1998). Relative strengths and weaknesses of small firms in innovation. *International Small Business Journal: Researching Entrepreneurship,**16*(3), 88–94. 10.1177/0266242698163005

[CR152] Wall, T., & Bellamy, L. (2019). Redressing small firm resilience: Exploring owner-manager resources for resilience. *International Journal of Organizational Analysis,**27*(2), 269–288. 10.1108/IJOA-02-2018-1364

[CR153] Welter, F., Schlepphorst, S., Schneck, S., & Holz, M. (2020). Der gesellschaftliche Beitrag des Mittelstands: Konzeptionelle Überlegungen. *Institut für Mittelstandsforschung (IfM) Bonn*, *IfM-Materialien*(283), 1–39.

[CR154] Werner, A., Schröder, C., & Chlosta, S. (2018). Driving factors of innovation in family and non-family SMEs. *Small Business Economics,**50*(1), 201–218. 10.1007/s11187-017-9884-4

[CR155] West, M. A., & Farr, J. L. (Eds.). (1996). *Innovation and creativity at work: Psychological and organizational strategies* (Repr). Wiley.

[CR156] WHO. (2020). *COVID-19 operationalization of the global response strategy in the WHO European Region*. WHO Regional Office for Europe.

[CR157] Wiklund, J., & Shepherd, D. (2003). Knowledge-based resources, entrepreneurial orientation, and the performance of small and medium-sized businesses. *Strategic Management Journal,**24*(13), 1307–1314. 10.1002/smj.360

[CR158] Wiklund, J., & Shepherd, D. (2005). Entrepreneurial orientation and small business performance: A configurational approach. *Journal of Business Venturing,**20*(1), 71–91. 10.1016/j.jbusvent.2004.01.001

[CR159] Williams, T. A., Gruber, D. A., Sutcliffe, K. M., Shepherd, D. A., & Zhao, E. Y. (2017). Organizational response to adversity: Fusing crisis management and resilience research streams. *Academy of Management Annals,**11*(2), 733–769. 10.5465/annals.2015.0134

[CR160] Wright, T. A. (2003). Positive organizational behavior: An idea whose time has truly come. *Journal of Organizational Behavior,**24*(4), 437–442. 10.1002/job.197

[CR161] Wright, T. A., & Quick, J. C. (2009). The role of positive-based research in building the science of organizational behavior. *Journal of Organizational Behavior,**30*(2), 329–336. 10.1002/job.581

[CR162] Wu, C.-M., & Chen, T.-J. (2018). Collective psychological capital: Linking shared leadership, organizational commitment, and creativity. *International Journal of Hospitality Management,**74*, 75–84. 10.1016/j.ijhm.2018.02.003

[CR163] Zhou, J., & George, J. M. (2001). When job dissatisfaction leads to creativity: Encouraging the expression of voice. *Academy of Management Journal,**44*(4), 682–696. 10.5465/3069410

